# Primary Bone Vascular Neoplasms: An Integrated Biological Perspective from Bone Pathology to Precision Oncology

**DOI:** 10.3390/cancers18193229

**Published:** 2026-10-07

**Authors:** Aikaterini Anetaki, Constantine Anetakis, Ioannis Chaniotakis, Christos Mpogiatzis, Anna Goussia, Dionysios J. Papachristou, Mattheos Bobos

**Affiliations:** 1 Department of Biomedical Sciences, School of Health Sciences, International Hellenic University, Sindos, 57400 Thessaloniki, Greecembobos@ihu.gr (M.B.); 2 Orthopaedic Department, University General Hospital of Alexandroupolis, 68100 Alexandroupolis, Greece; 3 Department of Pathology, Faculty of Medicine, School of Health Sciences, University of Ioannina, 45500 Ioannina, Greece; 4 Department of Anatomy, Histology and Embryology, School of Medicine, University of Patras, 26504 Patras, Greece; papachristoudj@med.upatras.gr

**Keywords:** primary bone vascular neoplasms, endothelial tumors, vascular anomalies, molecular pathology, bone microenvironment, osteoimmunology, precision oncology

## Abstract

Primary bone vascular neoplasms comprise a rare and biologically heterogeneous group of endothelial tumors encompassing benign, intermediate, and malignant entities with distinct clinicopathological, molecular, and prognostic characteristics. These tumors remain diagnostically and biologically challenging because of overlapping histopathological features, heterogeneous clinical behavior, evolving molecular landscapes, and limited disease-specific evidence. Advances in bone pathology, immunophenotyping, molecular diagnostics, and genomic profiling have substantially improved tumor classification and biological characterization. Importantly, contemporary classification and molecular studies have also highlighted the biological heterogeneity of vascular lesions involving bone, distinguishing well-defined vascular neoplasms from vascular malformations and identifying additional molecularly defined entities relevant to osseous disease and differential diagnosis. Concurrently, increasing recognition of the bone microenvironment and osteoimmunology provides a broader framework for understanding interactions among endothelial, skeletal, stromal, and immune populations and the extracellular matrix, with potential relevance to angiogenesis, bone remodeling, and tumor biology. Previous authoritative syntheses have established the classification, molecular features, diagnosis, and clinical management of these neoplasms. However, comparatively limited attention has been devoted to integrating tumor-intrinsic alterations with vascular–bone crosstalk, extracellular-matrix and mechanobiological signaling, osteoimmune regulation, and emerging precision-oncology strategies. Building on these foundations, this review integrates current evidence on classification, molecular pathogenesis, the skeletal microenvironment, osteoimmunology, biomarkers, and precision oncology. Where relevant, molecularly defined vascular neoplasms and vascular anomalies involving bone are discussed to clarify classification, molecular context, and differential diagnosis without conflating these lesions with the principal WHO-defined primary vascular neoplasms of bone. Findings directly demonstrated in primary bone vascular neoplasms are distinguished from mechanistic concepts extrapolated from physiological bone biology, other bone tumors, or non-osseous vascular neoplasms. Through this evidence-calibrated approach, we propose an integrated vascular–bone–immune framework, identify key knowledge gaps, and define priorities for disease-specific translational research and more individualized patient management.

## Simple Summary

Primary bone vascular neoplasms are rare endothelial tumors ranging from benign lesions to highly aggressive cancers. Their diagnosis is challenging because different entities may share similar microscopic features despite markedly different clinical behavior. Advances in molecular pathology have improved their classification, while contemporary classification systems have also emphasized the importance of distinguishing well-defined vascular tumors from vascular malformations and other vascular lesions involving bone. Growing knowledge of the bone microenvironment highlights interactions among vascular, bone, and immune cells. This review integrates contemporary pathological and molecular knowledge with emerging concepts in the bone microenvironment and osteoimmunology, providing a vascular–bone–immune framework for understanding these tumors.

## 1. Introduction

Primary bone vascular neoplasms comprise a rare and biologically heterogeneous group of endothelial tumors encompassing benign, intermediate, and malignant entities [1,2,3]. Importantly, these neoplastic entities should be distinguished from vascular malformations and complex vascular anomalies that may also involve bone and have historically been described using overlapping terminology.

Despite their rarity, these tumors present substantial diagnostic and therapeutic challenges because of their overlapping histopathological features, variable biological behavior, and the limited disease-specific evidence available to guide clinical management [3].

Advances in vascular tumor pathology have substantially improved the diagnosis and classification of these neoplasms. Historically, diagnosis relied primarily on histomorphological evaluation, which was frequently complicated by considerable overlap among benign, intermediate, and malignant vascular lesions. The incorporation of immunohistochemistry enabled more reliable confirmation of endothelial differentiation, whereas molecular pathology further refined classification through the identification of recurrent gene fusions and diagnostically informative genomic alterations. Contemporary diagnosis has therefore evolved from a predominantly morphology-based approach toward an integrated framework combining histopathology, immunophenotyping, targeted molecular testing, radiological findings, and clinical context [2,3,4,5,6].

Although these developments have improved diagnostic accuracy, tumor morphology and molecular alterations alone may not fully explain the biological heterogeneity of vascular neoplasms. Tumor development and progression are increasingly understood within the context of the specialized bone microenvironment, in which endothelial cells interact with osteoblasts, osteoclasts, osteocytes, mesenchymal stromal cells, immune populations, and the mineralized extracellular matrix. These interactions regulate angiogenesis, bone remodeling, and immune responses under physiological and pathological conditions and provide a biological framework for investigating how the skeletal microenvironment may influence primary bone vascular neoplasms [7,8,9,10,11,12].

This perspective is further informed by osteoimmunology, which examines the reciprocal interactions between the skeletal and immune systems under physiological and pathological conditions. Within the tumor microenvironment, immune–skeletal–vascular crosstalk may influence osteoclastogenesis, angiogenesis, inflammation, immune evasion, and disease progression, thereby extending current models beyond tumor cell-autonomous mechanisms [9,13,14,15,16].

Concurrently, advances in next-generation sequencing, multi-omics technologies, single-cell analysis, and spatial transcriptomics are enabling multidimensional characterization of bone tumors and may facilitate biomarker discovery, biological stratification, and the development of more precise diagnostic and therapeutic strategies [4,5,6,10,17,18,19,20,21,22].

Authoritative sources have established the contemporary classification of primary bone vascular neoplasms [1], while subsequent reviews have summarized the molecular genetics and diagnostic implications of vascular tumors across anatomical sites [2] and comprehensively synthesized the classification, clinicoradiological diagnosis, molecular features, and treatment of primary vascular tumors of bone [3]. These contributions provide essential diagnostic and clinical foundations. However, comparatively limited attention has been devoted to integrating tumor-intrinsic molecular alterations with the specialized skeletal vascular niche, bone remodeling, extracellular-matrix and mechanobiological signaling, osteoimmune regulation, and emerging precision-oncology strategies.

Building on this foundation, the present review adopts an integrated vascular–bone–immune framework, with explicit attention to the source and strength of the available evidence. It connects contemporary pathological and molecular findings with vascular–bone crosstalk, the bone microenvironment, and osteoimmunology, and examines how these interactions may inform biomarker development, biological stratification, and precision-oncology research. Findings directly demonstrated in primary bone vascular neoplasms are distinguished from mechanistic concepts extrapolated from physiological bone biology, other bone tumors, or non-osseous vascular neoplasms, as well as from molecular findings derived from vascular anomalies and other vascular lesions that may involve bone. Through this approach, the review identifies current knowledge gaps and defines priorities for disease-specific translational investigation and more individualized patient management.

## 2. Materials and Methods

This review was designed as an integrative narrative synthesis of the current evidence on primary bone vascular neoplasms.

A targeted literature search supporting this integrative narrative review was conducted using PubMed/MEDLINE, Scopus, and Google Scholar. Search terms included combinations of “primary bone vascular neoplasms”, “vascular tumors of bone”, “intraosseous vascular tumors”, “bone hemangioma”, “epithelioid hemangioma”, “epithelioid hemangioendothelioma”, “angiosarcoma of bone”, “pseudomyogenic hemangioendothelioma”, “spindle cell hemangioma”, “complex lymphatic anomaly”, “vascular malformation”, “intraosseous venous malformation”, “vertebral hemangioma”, “intraosseous hemangioma”, and “Kaposi sarcoma”, combined where appropriate with “bone”, “osseous”, or “intraosseous”. Molecular searches included “*FOS*”, “*FOSB*”, “*WWTR1*::*CAMTA1*”, “*YAP1*::*TFE3*”, “*EWSR1*::*NFATC2*”, “*NFATC1*”, “*NFATC2*”, “*SERPINE1*::*FOSB*”, “*ACTB*::*FOSB*”, “*GNAQ*”, “*GNA11*”, “*IDH1*”, “*IDH2*”, “*PIK3CA*”, “*PTEN*”, “*TEK*”, “*ELMO2*”, “GLUT1”, “D2-40”, “*podoplanin*”, “*KRAS*”, “*MYC*”, “*FLT4*”, and “*KDR*”. Additional targeted searches addressed “molecular pathology”, “bone microenvironment”, “angiogenesis”, “osteoimmunology”, “endothelial biology”, “tumor microenvironment”, “biomarkers”, “multi-omics”, “single-cell and spatial profiling”, “artificial intelligence”, “precision oncology”, and “emerging therapeutic strategies” when relevant to the translational scope of the review. The literature search was last updated in September 2026.

Priority was given to authoritative classification systems and consensus recommendations, original clinicopathological and molecular studies, disease-specific cohort studies, and mechanistic or translational investigations directly relevant to the biological framework of the review. Recent high-quality review articles were also considered when they provided comprehensive syntheses of rapidly evolving areas, including molecular pathology, vascular and bone biology, osteoimmunology, tumor–microenvironment interactions, and precision oncology. No formal publication-date restriction was applied; however, emphasis was placed on literature published during the most recent five-year period, while earlier seminal and landmark studies were retained when essential for establishing disease classification, defining recurrent molecular alterations, or providing foundational mechanistic evidence. Given the exceptional rarity of primary bone vascular neoplasms, evidence was not restricted to prospective studies or large cohorts; retrospective series, molecularly characterized case series, and selected studies of related vascular neoplasms were considered when they provided relevant diagnostic, biological, or translational information.

The selected evidence was organized into complementary domains encompassing clinicopathological classification and differential diagnosis, molecular alterations and tumor pathogenesis, vascular–bone and immune–skeletal interactions, diagnostic and emerging biomarkers, and current or investigational therapeutic strategies. The level and origin of the available evidence were explicitly considered. Findings directly demonstrated in primary bone vascular neoplasms were distinguished from mechanistic concepts derived from physiological bone biology, osteoimmunology, or other tumor systems. Evidence derived from vascular malformations involving bone was additionally distinguished from that pertaining to primary vascular neoplasms, particularly where historical terminology such as “hemangioma” overlaps with contemporary vascular-anomaly classification. Given the narrative and integrative scope of the review, together with the rarity and heterogeneity of the available evidence, no formal meta-analysis or quantitative evidence synthesis was performed.

OpenAI ChatGPT (GPT-5.6 Sol; OpenAI, San Francisco, CA, USA; accessed August–September 2026) was used solely for image editing and graphical refinement, including preparation of the graphical abstract. No artificial intelligence tool was used to generate or analyze study data. All outputs were critically reviewed and edited by the authors, who take full responsibility for the content of the publication.

**Figure 1:**
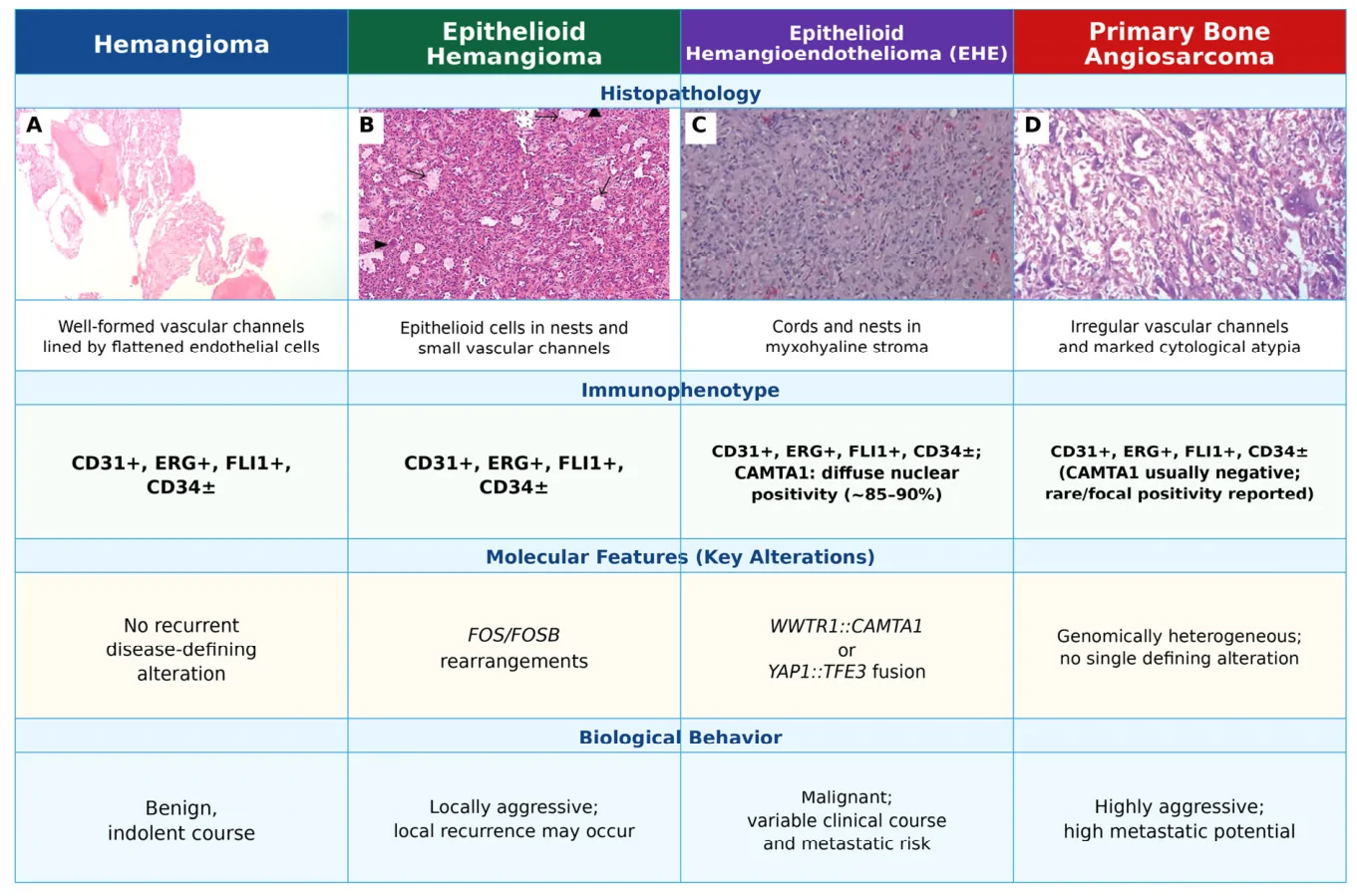
Representative histopathological, immunophenotypic, molecular, and biological features of primary bone vascular neoplasms. Representative histopathological features of (**A**) hemangioma of bone, characterized by well-formed vascular channels lined by cytologically bland, flattened endothelial cells; (**B**) epithelioid hemangioma, showing plump epithelioid endothelial cells associated with well-formed vascular spaces; black arrowheads indicate representative foci of epithelioid endothelial cells; (**C**) epithelioid hemangioendothelioma (EHE), characterized by epithelioid endothelial cells arranged in cords and nests within a myxohyaline stroma, with variable intracytoplasmic vacuolization; and (**D**) primary bone angiosarcoma, showing malignant endothelial proliferation with cytological atypia and irregular vasoformative architecture. The principal immunophenotypic, molecular, and biological features of each entity are summarized below the corresponding histological panel [1,3,35,47,50,54,55,64]. Diffuse nuclear CAMTA1 expression is observed in approximately 85–90% of EHEs and is typically associated with the WWTR1::CAMTA1 fusion. Other vascular tumors are generally CAMTA1-negative, although rare or focal CAMTA1 immunoreactivity has been reported, including in angiosarcoma. Histopathological image (**A**) was provided by co-author D. J. Papachristou. Images were adapted from (**B**) Sin et al. [65], Figure 5b; (**C**) Plumby et al. [66], Figure 5; and (**D**) Chugh et al. [67], Figure 3b.

Representative histopathological images included in Figure 1 were either provided by a co-author or obtained from previously published open-access sources and were selected to illustrate the characteristic morphological features of the corresponding vascular neoplasms. Image (A) was provided by co-author D. J. Papachristou, whereas images (B–D) were adapted from previously published sources, as specified individually in the Figure 1 legend. The images were cropped, resized, and reformatted solely for graphical consistency, without alteration of the underlying histopathological content.

## 3. Classification, Epidemiology, and Clinical Presentation

### 3.1. Contemporary Classification

The classification of primary bone vascular neoplasms has evolved substantially with advances in molecular pathology and integrated diagnostic approaches. Previously defined predominantly by histomorphology, these rare endothelial tumors are now recognized as biologically distinct entities characterized by specific clinicopathological and, in selected cases, molecular features [1,2,4,5,18,23].

The WHO Classification of Soft Tissue and Bone Tumors recognizes four primary vascular neoplasms of bone—hemangioma, epithelioid hemangioma, epithelioid hemangioendothelioma, and angiosarcoma—representing biologically distinct clinicopathological entities rather than sequential stages of tumor progression [1,2,3,23]. Although all four entities share endothelial differentiation, they differ substantially in biological behavior, recurrence risk, metastatic potential, clinical outcome, and therapeutic implications [3]. Importantly, recurrent molecular alterations have contributed to the definition of selected entities and facilitate diagnostic distinction in morphologically challenging cases [1,2,3,23].

An important distinction exists between the WHO Classification of Soft Tissue and Bone Tumors [1,24] and the International Society for the Study of Vascular Anomalies (ISSVA) Classification [25,26,27]. Whereas the WHO framework focuses primarily on the pathological, immunophenotypic, molecular, and biological characterization of vascular neoplasms, including primary bone vascular neoplasms, the ISSVA Classification encompasses the broader spectrum of vascular anomalies and distinguishes vascular tumors from vascular malformations while increasingly incorporating genetic and molecular information into disease classification and nomenclature [1,24,25,26,27]. The two systems therefore differ in scope and primary purpose but provide complementary perspectives. For primary bone vascular neoplasms, the WHO framework remains the principal pathological classification system [1,24], whereas the ISSVA framework is particularly relevant for distinguishing true vascular neoplasms from developmental vascular anomalies and malformations [25,26,27]. The principal differences between the two classification frameworks are summarized in Table 1.

#### Hemangioma of Bone: Terminological and Biological Heterogeneity

The term hemangioma of bone has historically been applied to a heterogeneous spectrum of intraosseous vascular lesions and should therefore not be assumed to represent a single biological entity. This distinction is particularly important when reconciling the World Health Organization (WHO) classification of bone tumors with the International Society for the Study of Vascular Anomalies (ISSVA) classification. These systems are complementary but serve different purposes: the WHO provides a pathology-based framework for the classification of bone neoplasms, whereas the ISSVA fundamentally distinguishes vascular anomalies into vascular tumors and vascular malformations [1,25,26].

Importantly, the updated ISSVA 2025 terminology further emphasizes this distinction: lesions historically designated as “intraosseous hemangioma” are considered to represent venous malformations in most instances and are more appropriately designated intraosseous venous malformations within the ISSVA framework [1,25,26]. This terminology should not, however, be interpreted as demonstrating that every lesion historically diagnosed as hemangioma of bone necessarily represents a venous malformation; rather, it highlights the biological heterogeneity encompassed by historical nomenclature.

Application of the ISSVA framework to osseous vascular lesions has demonstrated that historical terminology does not always correspond to contemporary biological classification. In a clinicopathological reappraisal of 77 vascular lesions of bone, 46 were classified as vascular malformations and 31 as vascular tumors [28]. This terminological discrepancy is particularly relevant to lesions historically described as calvarial hemangioma, intraosseous hemangioma, or intraosseous cavernous hemangioma, some of which—particularly those involving the calvaria, skull base, and facial skeleton—are more appropriately classified as non-neoplastic intraosseous venous malformations [28,29]. Additional bone-specific evidence supports this distinction. Kadlub et al. re-evaluated three pediatric jaw lesions previously diagnosed as intraosseous hemangiomas and demonstrated that, although they could be classified as central hemangiomas according to the WHO framework, they fulfilled ISSVA criteria for slow-flow vascular malformations; all three lesions were GLUT1-negative and D2-40-negative [30]. Similarly, a series of eight craniofacial intraosseous venous malformations documented lesions that had previously been inappropriately designated as hemangiomas and emphasized integration of cross-sectional imaging, histopathology, and immunohistochemistry, including GLUT1, for accurate classification [31]. Consistent with this evolving terminology, a recent series of facial intraosseous vascular anomalies emphasized that many intraosseous vascular malformations have historically been mislabeled as “hemangiomas” and supported classification within the contemporary vascular-anomaly framework rather than reliance on traditional intraosseous hemangioma terminology [32]. More recently, a 2025 systematic review of maxillary venous malformations highlighted this nomenclatural transition, using the term venous malformation for lesions historically described as cavernous hemangiomas [33].

The established term vertebral hemangioma nevertheless remains widely used in the clinical and radiological literature and is retained here where appropriate, without implying that all intraosseous vascular lesions designated as “hemangiomas” share the same biological nature.

Current evidence is insufficient to establish that vascular lesions historically termed vertebral, calvarial, craniofacial, jaw, and appendicular bone hemangiomas constitute a single biologically or molecularly uniform entity. Conversely, available evidence also does not justify reclassifying every lesion historically diagnosed as conventional hemangioma of bone as a venous malformation. Anatomical site, imaging characteristics, histopathology, clinical behavior, and, where informative, molecular findings should therefore be integrated when assigning the most appropriate diagnosis.

Importantly, lesions historically designated as conventional hemangioma of bone should not be regarded as direct osseous counterparts of cutaneous infantile hemangioma. Infantile hemangioma is a clinically and biologically defined vascular tumor characterized by a proliferative phase followed by spontaneous involution and by a characteristic GLUT1 immunophenotype, whereas comparable defining biological and immunophenotypic criteria have not been established for conventional hemangioma of bone [34]. Thus, the shared term “hemangioma” should not be interpreted as evidence of biological equivalence between these lesions.

Historical morphological qualifiers also require caution. Terms such as “cavernous” and “capillary” have been used descriptively for intraosseous vascular lesions but should not be assumed to define biologically equivalent neoplastic subtypes within a single bone-hemangioma spectrum. In contrast, epithelioid hemangioma and spindle cell hemangioma represent distinct vascular-tumor entities with characteristic clinicopathological and, in selected cases, molecular features, including recurrent FOS/FOSB rearrangements in epithelioid hemangioma and IDH1/IDH2 alterations in spindle cell hemangioma [35,36,37]. These molecularly characterized entities should therefore not be conflated with historically designated conventional, cavernous, or capillary intraosseous hemangiomas solely because they share the term “hemangioma.”

GLUT1 may provide useful ancillary information in this differential diagnosis but should not be interpreted as a stand-alone discriminator. A systematic review found GLUT1 positivity in 368 of 386 (95%) infantile hemangiomas, whereas all 54 venous malformations evaluated were GLUT1-negative; importantly, congenital hemangiomas were also GLUT1-negative, demonstrating that GLUT1 is not a universal marker of vascular tumors designated as hemangiomas [34]. Accordingly, GLUT1 negativity is not specific for venous malformation, and bone-specific validation remains limited. The diagnostic value of GLUT1 in intraosseous lesions should therefore be interpreted in conjunction with morphology, imaging, anatomical site, and clinical context [30,31,34].

Molecular findings provide an additional level of biological discrimination. Somatic activating TEK mutations are established drivers of sporadic venous malformations [38], while activating PIK3CA mutations account for an additional molecular subset of venous malformations, including 27 of 50 (54%) TEK-negative lesions in the series of Limaye et al. [39]. These alterations converge on PI3K–AKT signaling and support venous-malformation biology when identified in an appropriately classified lesion. However, these molecular data derive predominantly from venous malformations in general rather than from large, systematically characterized series of intraosseous lesions. TEK and PIK3CA alterations have not been systematically demonstrated across conventional hemangiomas of bone, and their absence does not exclude a venous malformation. Accordingly, these molecular findings should not be extrapolated as defining features of conventional bone hemangioma.

Of particular relevance to osseous disease, a recent report identified a somatic PIK3CA mutation in a recurrent vertebral venous malformation, providing direct evidence that at least some vertebral vascular lesions may show PIK3CA-driven venous-malformation biology [40]. This observation is important but currently represents case-level evidence and should not be generalized to vertebral hemangiomas as a group. Furthermore, biallelic loss-of-function variants in ELMO2 define an autosomal-recessive form of intraosseous vascular malformation (VMOS), initially demonstrated in five families [41], with subsequent additional cases further expanding the clinical and molecular spectrum of ELMO2-related intraosseous vascular malformation [42]. These findings provide direct evidence that genetically distinct vascular malformations can primarily involve bone and should not be conflated with conventional hemangioma of bone.

Similar caution is required for the historical terms skeletal angiomatosis, cystic angiomatosis, and lymphangiomatosis, which should not be equated with multifocal bone hemangioma. Within the contemporary ISSVA framework, complex lymphatic anomalies include distinct entities such as generalized lymphatic anomaly (GLA), Gorham–Stout disease (GSD), and kaposiform lymphangiomatosis (KLA). GLA typically shows multifocal osseous involvement, whereas GSD is characterized by progressive osteolysis with cortical bone destruction [26,43].

Despite this terminological and biological heterogeneity, hemangioma of bone remains an established benign entity within the pathology-based classification of primary vascular tumors of bone. In a morphologic and molecular analysis of 427 primary vascular bone tumors, conventional hemangiomas represented the largest diagnostic category (289/427 cases), and both conventional and epithelioid hemangiomas showed benign clinical behavior [44]. This large series supports the continued use of hemangioma as a pathology-based diagnostic designation but does not establish that all lesions included under this historical term constitute a single molecularly defined neoplastic entity.

Accordingly, throughout this review, hemangioma of bone is retained when used within the appropriate WHO/pathology-based context, but not as an umbrella term for all benign intraosseous vascular lesions. Whenever supported by clinicopathological evidence, vascular neoplasms are distinguished from vascular malformations and the most specific entity designation is used. The principal clinicopathological, immunophenotypic, and molecular features distinguishing conventional hemangioma of bone from intraosseous venous malformation are summarized in Table 2. Where classification remains uncertain or historical terminology differs from contemporary ISSVA terminology, both the original designation and its contemporary biological interpretation should be explicitly acknowledged. The molecular heterogeneity underlying these vascular tumors and malformations, including entity-specific genetic alterations that further refine their biological classification, is discussed in detail in the molecular pathology section below.

### 3.2. Epidemiology

Primary bone vascular neoplasms constitute a small proportion of primary skeletal tumors and are rare entities in musculoskeletal pathology. Their true incidence remains difficult to determine because current evidence derives predominantly from retrospective institutional series, registry-based studies, and relatively small clinicopathological cohorts. This uncertainty reflects not only the rarity of these tumors but also evolving pathological classification and the incorporation of molecular diagnostics, which have refined disease definitions and complicate direct comparisons between historical and contemporary series [1,2,3].

Despite their common endothelial origin, individual entities exhibit distinct epidemiological characteristics. Lesions historically designated as intraosseous hemangiomas are most frequently encountered in the vertebral column and craniofacial skeleton; however, this terminology encompasses biologically heterogeneous vascular lesions, and some craniofacial lesions are more appropriately classified as intraosseous venous malformations, as discussed above.

Epithelioid hemangioma (EH) commonly involves the long bones of young and middle-aged adults and may present as multifocal disease [1,2,3,35]. Epithelioid hemangioendothelioma (EHE) demonstrates variable clinical behavior; multifocal skeletal involvement is well recognized and may mimic metastatic bone disease [1,2,3,46]. In contrast, primary angiosarcoma of bone predominantly affects adults and remains a rare and highly aggressive vascular neoplasm of the skeleton [1,2,3,47]. Recognition of these entity-specific epidemiological patterns strengthens clinicopathological correlation and contributes to integrated diagnostic assessment.

### 3.3. Clinical Presentation and Diagnostic Implications

Clinical manifestations are influenced primarily by biological behavior and anatomical location rather than endothelial differentiation itself. Benign vascular neoplasms are frequently asymptomatic or present with nonspecific localized pain, whereas more aggressive tumors are more commonly associated with persistent pain, swelling, cortical destruction, pathological fracture, soft-tissue extension, and functional impairment. Importantly, clinicoradiological aggressiveness does not always parallel histological classification, highlighting the biological heterogeneity of primary bone vascular neoplasms [1,2,3].

This concept is particularly evident among epithelioid vascular tumors. Epithelioid hemangioma may exhibit locally aggressive radiological features, including cortical destruction and extension into adjacent soft tissues, despite its generally favorable biological behavior [1,2,3,35,48,49]. Conversely, epithelioid hemangioendothelioma may demonstrate relatively bland histopathological features while retaining the capacity for multifocal skeletal involvement, local recurrence, and distant metastasis [1,2,3,46]. Angiosarcoma typically presents as an aggressive destructive lesion and is associated with a substantial risk of metastatic dissemination [1,2,3,47,50]. Collectively, these observations emphasize that neither clinical presentation nor imaging findings alone reliably predict biological behavior or establish a definitive diagnosis [1,2,3].

Imaging remains indispensable for lesion detection, characterization, local staging, and surgical planning but generally lacks sufficient specificity for definitive classification. Radiological findings should therefore be interpreted in conjunction with clinical, histopathological, immunophenotypic, and molecular findings [1,2,48]. This clinicopathological heterogeneity underscores the importance of multidisciplinary assessment, particularly for lesions with overlapping morphological or radiological features, to achieve accurate classification and guide appropriate clinical management [1,2,3].

## 4. Histopathological Features and Differential Diagnosis

### 4.1. Histopathology as the Diagnostic Cornerstone

Histopathological evaluation remains the cornerstone of diagnosis in primary bone vascular neoplasms, but morphology must be interpreted within the clinicoradiological context. Diagnostic assessment should consider vascular architecture, endothelial cytomorphology, degree of vasoformation, growth pattern, stromal composition, mitotic activity, necrosis, and the relationship of the lesion to pre-existing bone. No single feature reliably separates benign, intermediate, and malignant tumors, and substantial overlap is particularly evident among epithelioid vascular neoplasms. Contemporary pathological diagnosis therefore depends on recognizing reproducible combinations of architectural, cytological, immunophenotypic, and molecular features rather than relying on an isolated “classic” feature [1,3,5,9,15,18].

Recent advances in artificial intelligence-assisted image analysis, multimodal deep-learning approaches, and molecular diagnostics are expanding the tools available for integrated tumor assessment [51,52,53]. Emerging AI-based approaches in bone tumors illustrate the potential integration of imaging, pathological, clinical, and biomarker data to support diagnostic assessment, although their application remains evolving and requires further validation [51,52,53]. Expert morphological assessment, integrated with immunophenotypic and molecular findings, nevertheless remains fundamental to definitive pathological diagnosis [4,18,51,52,53]. The principal histopathological, immunophenotypic, molecular, and diagnostic features of primary bone vascular neoplasms recognized in the WHO 2020 classification are summarized in Table 3.

### 4.2. Histopathological Spectrum and Differential Diagnosis

Within the WHO/pathology-based framework, lesions classified under the conventional hemangioma of bone designation are benign vascular lesions typically composed of well-formed vascular channels lined by flattened endothelial cells lacking significant cytological atypia, mitotic activity, or destructive infiltration. As discussed above, this pathological designation should be distinguished from the heterogeneous group of intraosseous vascular malformations and complex vascular anomalies that have historically also been described using the term “hemangioma.” Accordingly, the term conventional hemangioma of bone is used here in its WHO/pathology-based context and should not be interpreted as implying that all intraosseous lesions historically designated as “hemangiomas” represent a single biologically or molecularly uniform neoplastic entity [1,25,26,28,29,44].

Its orderly vasoformative architecture generally allows distinction from malignant vascular tumors [1,44], although limited biopsy material, thrombosis, secondary hemorrhage, or reactive osseous change may obscure the underlying pattern [1,2,50]. Distinction from an intraosseous venous malformation may be more challenging because bland vascular morphology can overlap and histomorphology alone may not reliably resolve the distinction in all historically designated “hemangiomas.” In such cases, histopathological findings should be integrated with anatomical location, clinical evolution, and characteristic radiological features, together with ancillary immunohistochemical findings where informative [28,29,30,31]. GLUT1 may contribute to this integrated assessment, particularly when an infantile-hemangioma phenotype is considered, but GLUT1 negativity alone should not be interpreted as diagnostic of venous malformation [34]. Epithelioid hemangioma (EH) is more diagnostically challenging. It contains plump epithelioid endothelial cells with eosinophilic cytoplasm, intracytoplasmic vacuoles, and variable formation of small vascular lumina, frequently accompanied by a prominent inflammatory infiltrate. Cellular areas, nuclear atypia, necrosis, cortical destruction, and soft-tissue extension may create an alarming appearance despite the tumor’s generally favorable behavior. In a molecularly confirmed series of 38 skeletal cases, regional multifocality and destructive radiological features contributed to frequent initial misclassification as another vascular tumor [35]. These findings highlight epithelioid hemangioma as an important source of diagnostic overcalling within the skeletal epithelioid vascular spectrum.

Conventional epithelioid hemangioendothelioma is characterized by cords, short strands, or small nests of epithelioid endothelial cells embedded within a myxohyaline or densely hyalinized stroma. Intracytoplasmic vacuoles, occasionally containing erythrocytes, provide evidence of primitive endothelial differentiation, whereas fully developed vascular channels are usually inconspicuous. Cytological atypia and mitotic activity may remain limited despite the capacity for multifocal disease, recurrence, and metastasis. This discordance between relatively bland morphology and clinically meaningful malignant potential represents an important diagnostic feature of EHE. International consensus recommendations recognize pathological examination and molecular confirmation as central to reliable diagnosis, particularly in anatomically or morphologically atypical lesions [3,46,54,55]. Recognition of the characteristic myxohyaline matrix remains particularly valuable in small biopsies, where cytological atypia alone may underestimate biological potential.

*YAP1::TFE3*-fused hemangioendothelioma shows a partially overlapping but distinctive phenotype. Tumors frequently contain larger epithelioid cells with abundant eosinophilic or vacuolated cytoplasm, prominent nucleoli, solid or pseudoalveolar areas, and more evident vasoformation than conventional EHE. Multi-institutional studies have demonstrated considerable architectural heterogeneity and support recognition of *YAP1::TFE3*-fused hemangioendothelioma as a distinct molecularly defined subset within the broader spectrum of epithelioid vascular tumors [54,59,60,61].

Angiosarcoma represents the malignant extreme and shows infiltrative, irregular, anastomosing vascular channels or solid sheets of markedly atypical endothelial cells. Features supporting malignancy include destructive permeation of bone, pronounced nuclear pleomorphism, multilayering or tufting of endothelial cells, brisk mitotic activity, atypical mitoses, necrosis, and loss of recognizable vasoformation in poorly differentiated areas. Primary angiosarcoma of bone may nevertheless be morphologically heterogeneous, and small biopsies can sample deceptively differentiated or relatively bland regions. A recent bone-specific series documented frequent multifocal disease, advanced presentation, and aggressive clinical outcomes, reinforcing the need to integrate histological findings with complete radiological staging and adequate tissue sampling [1,47,50,54]. Marked intratumoral heterogeneity further contributes to sampling bias in limited biopsies.

The most difficult distinction is frequently between epithelioid hemangioma and epithelioid hemangioendothelioma. Both may display epithelioid cytology, intracytoplasmic vacuoles, limited atypia, and multifocal skeletal involvement. Well-developed vasoformation, lobular architecture, and a conspicuous inflammatory component favor epithelioid hemangioma, whereas cords and nests embedded within myxohyaline stroma favor EHE. However, these criteria are not uniformly expressed, particularly in cellular EH or limited biopsies. Demonstration of *FOS* or *FOSB* rearrangements supports EH, whereas *WWTR1::CAMTA1* or *YAP1::TFE3* confirms the corresponding hemangioendothelioma subtype. Rare fusion partners and atypical morphology further illustrate why molecular findings must be interpreted in conjunction with morphology rather than used as isolated diagnostic labels [3,35,54,55].

Distinguishing EHE from angiosarcoma requires assessment of the entire morphological pattern rather than reliance on epithelioid cytology alone. Myxohyaline stroma, intracytoplasmic lumina, limited pleomorphism, and relatively low mitotic activity support conventional EHE. In contrast, marked atypia, destructive vasoformation, high proliferative activity, atypical mitoses, and extensive necrosis favor angiosarcoma. Nevertheless, atypical EHE may exhibit increased cellularity, solid growth, necrosis, and elevated mitotic activity, thereby approaching angiosarcoma morphologically. Molecular confirmation is therefore particularly important in morphologically ambiguous or high-grade-appearing lesions [46,47,50].

Epithelioid vascular tumors must also be distinguished from metastatic carcinoma, melanoma, epithelioid sarcoma, and other epithelioid malignancies involving bone. Focal cytokeratin expression can occur in EHE and angiosarcoma and should not automatically imply epithelial differentiation. Conversely, CD31 and ERG provide strong evidence of endothelial lineage, although no immunohistochemical marker should be interpreted independently of morphology. Melanocytic markers help exclude melanoma, while loss of INI1 supports epithelioid sarcoma in the appropriate context. Pseudomyogenic hemangioendothelioma is another relevant mimic, particularly in younger patients with multifocal extremity disease; its fascicular eosinophilic cells, frequent keratin expression, and characteristic *FOSB*-associated biology distinguish it from primary epithelioid vascular neoplasms of bone [46,50,54,62,63].

Reactive vascular proliferations and vascular malformations may simulate benign neoplasia, especially in fragmented or hemorrhagic specimens. Preservation of normal tissue relationships, absence of a destructive infiltrative pattern, and lack of significant endothelial atypia favor a non-neoplastic process. Clinicoradiological correlation is essential because reactive changes adjacent to fracture, hemorrhage, or another primary bone lesion may otherwise be overinterpreted as a vascular tumor [1,3].

### 4.3. Integrated Pathological Diagnosis

Immunohistochemistry refines rather than replaces morphological diagnosis. CD31 and ERG remain the most reliable markers for establishing endothelial differentiation, while CD34 and FLI1 provide supportive but less specific information. Nuclear CAMTA1 supports conventional *WWTR1::CAMTA1*-positive EHE, whereas TFE3 and loss of YAP1 C-terminal expression may assist in recognizing *YAP1::TFE3*-fused tumors. FOS and FOSB expression may support epithelioid hemangioma, but expression patterns are not completely specific and discordant cases require molecular confirmation. Surrogate immunohistochemical markers should therefore be interpreted cautiously, particularly TFE3 and FOSB, because expression may extend beyond a single molecular entity [3,5,18,63].

The contemporary diagnostic pathway is hierarchical but integrated: morphology defines the differential diagnosis, immunophenotyping establishes endothelial lineage and may identify potential molecular subtypes, and targeted molecular testing resolves diagnostically ambiguous or clinically consequential cases. Recurrent genetic alterations therefore provide diagnostically defining or highly supportive information in selected entities rather than serving merely as ancillary findings [4,5,51].

Next-generation sequencing (NGS) and RNA sequencing may provide additional support in diagnostically challenging tumors, particularly for the identification of uncommon fusions or rare molecular subtypes. Emerging approaches, including DNA methylation profiling, digital pathology, and computational image analysis, may further improve diagnostic reproducibility, while complementing rather than replacing expert histopathological interpretation [4,5,18,51,52,53]. These integrated pathological features are summarized in Figure 1 and provide the basis for examining the molecular mechanisms underlying the biological diversity of primary bone vascular neoplasms.

## 5. Molecular Pathology and Pathogenesis

### 5.1. Molecular Landscape of Primary Bone Vascular Neoplasms

Molecular characterization has revealed that primary bone vascular neoplasms arise through biologically distinct oncogenic mechanisms. Epithelioid hemangioma and epithelioid hemangioendothelioma are characterized by recurrent gene fusions that define major molecular subsets, whereas primary bone angiosarcoma exhibits a substantially more heterogeneous genomic landscape without a single disease-defining molecular alteration [1,2,35,47,50,54,55,64].

In molecular subsets of epithelioid hemangioma, recurrent *FOS* and *FOSB* rearrangements support clonal endothelial neoplasia and implicate AP-1 family transcriptional dysregulation [35,36,68]. Conventional epithelioid hemangioendothelioma is characterized by the highly recurrent *WWTR1::CAMTA1* fusion, whereas a distinct molecular subset harbors *YAP1::TFE3*, linking EHE pathogenesis to dysregulated Hippo-pathway transcriptional control [54,55,59,60,61].

The molecular spectrum of vascular lesions involving bone is, however, broader than the canonical alterations identified in epithelioid hemangioma and epithelioid hemangioendothelioma. Molecular studies increasingly demonstrate that lesions historically grouped under descriptive terms such as hemangioma, angiomatosis, or related vascular proliferations may represent biologically distinct neoplastic or malformative processes. These findings are particularly relevant to the distinction between vascular tumors and vascular malformations discussed above and indicate that molecular alterations should be interpreted within their specific clinicopathological and anatomical context rather than extrapolated across all osseous vascular lesions.

An emerging molecularly defined group comprises vascular neoplasms with *NFATC1* or *NFATC2* rearrangements. An *EWSR1::NFATC1* fusion was initially described in a benign vascular tumor of bone [69] and subsequent studies have expanded this spectrum to include *EWSR1::NFATC1*, *EWSR1::NFATC2*, *FUS::NFATC2*, and other less common fusion partners. In a recent series of 14 NFATC-altered vascular neoplasms, 12 arose in bone, supporting the existence of a predominantly intraosseous group with overlapping morphological features [70]. Importantly, NFATC-family rearrangements are not exclusively restricted to vascular neoplasms and have also been identified in other bone lesions and sarcomas; consequently, their diagnostic significance depends on integration with morphology, radiology, immunophenotype, and broader molecular context.

Additional molecular alterations further illustrate the heterogeneity of vascular proliferations involving bone. A somatic *GNAQ* p.T96S mutation has been demonstrated in diffuse bone and soft-tissue angiomatosis [71], providing direct evidence that Gαq-pathway alterations may occur in selected osseous vascular lesions. This observation should not, however, be generalized to conventional hemangioma of bone, and current bone-specific evidence for *GNA11* alterations remains substantially more limited. Spindle cell hemangioma represents another molecularly distinctive vascular neoplasm, characterized by recurrent hotspot mutations in *IDH1* and *IDH2*. Kurek et al. identified *IDH1/IDH2* mutations in 20 of 28 spindle cell hemangiomas (71%), predominantly *IDH1* p.R132C, while *IDH1* p.R132C was absent from 154 other vascular tumors and malformations examined in the same study [37]. These alterations therefore provide strong entity-associated molecular support for spindle cell hemangioma but should not be interpreted as a general molecular signature of osseous hemangiomas.

Molecular characterization has also substantially refined the understanding of complex vascular and lymphatic malformations with skeletal involvement. Somatic activating *PIK3CA* mutations have been demonstrated in generalized lymphatic anomaly (GLA), including patients with multifocal skeletal disease, implicating constitutive PI3K–AKT–mTOR signaling in its pathogenesis [72]. Somatic activating *KRAS* variants have similarly been identified in Gorham–Stout disease (GSD), with experimental evidence supporting a mechanistic relationship between activated RAS–MAPK signaling, abnormal lymphatic development, and osseous involvement [73,74]. In the broader vascular-malformation spectrum, somatic *TEK* mutations constitute established drivers of venous malformations [38], while somatic activating PIK3CA mutations define an additional major molecular subset. Limaye et al. identified PIK3CA mutations in 27 of 50 (54%) venous malformations lacking detectable TEK mutations, with both TEK- and PIK3CA-driven lesions converging on dysregulated PI3K–AKT signaling [39]. Although most molecular data on TEK- and PIK3CA-driven venous malformations are not bone-specific, recent evidence demonstrates that this molecular framework can also apply to selected intraosseous lesions. A recurrent vertebral venous malformation harboring a somatic PIK3CA p.Glu542Lys mutation was recently reported, providing direct evidence of PIK3CA-driven venous-malformation biology within the vertebral compartment [40]. This observation currently represents case-level evidence and should not be generalized to lesions conventionally classified as vertebral hemangiomas.

A distinct genetically defined intraosseous vascular-malformation disorder is primary intraosseous vascular malformation associated with biallelic loss-of-function variants in ELMO2. The disorder was initially characterized in five families, providing direct evidence for a genetically defined inherited vascular malformation with prominent osseous involvement [41]. More recent additional cases with novel pathogenic ELMO2 variants have further expanded its clinical and molecular spectrum [42]. ELMO2-related intraosseous vascular malformation therefore provides further evidence that molecularly distinct vascular malformations may primarily involve bone and should not be conflated with conventional hemangioma of bone.

Vascular anomalies may also occur within the phenotypic spectrum of germline *PTEN* alterations [75]. Collectively, these findings demonstrate that molecularly defined vascular malformations involving bone comprise biologically distinct entities and pathways rather than a single “hemangioma” spectrum. These molecular findings should be attributed to their respective malformation syndromes and should not be extrapolated to conventional primary hemangioma of bone.

Pseudomyogenic hemangioendothelioma (PHE), which may involve bone as part of multifocal or occasionally predominantly osseous disease [76], is characterized by recurrent *FOSB*-activating rearrangements. The canonical *SERPINE1::FOSB* fusion was identified as a recurrent molecular event in PHE [77], while *ACTB::FOSB* and rarer *FOSB* fusion partners have subsequently expanded its molecular spectrum [78,79]. FOSB immunohistochemistry provides a useful diagnostic surrogate [62], although expression is not entirely specific and molecular confirmation may be valuable in morphologically ambiguous lesions. Thus, PHE represents a further example in which fusion-defined biology can refine the classification of vascular neoplasms involving bone.

Broader genomic studies of angiosarcoma have identified heterogeneous alterations affecting angiogenic signaling, proliferative and cell-survival pathways, and genomic stability [47,80,81,82]. Bone-specific studies further confirm substantial intertumoral genomic heterogeneity, including *MYC* amplification in a subset of primary angiosarcomas of bone [57]. Importantly, *MYC* amplification is substantially enriched in secondary angiosarcoma, while *FLT4* amplification has been reported predominantly in secondary, frequently *MYC*-amplified disease [56,58]. These etiological differences and their implications for primary bone angiosarcoma are discussed in greater detail in Section 5.3.

Kaposi sarcoma represents a biologically distinct HHV-8/KSHV-associated vascular neoplasm that may rarely involve bone but should not be classified among the conventional primary vascular neoplasms of bone. Musculoskeletal involvement is uncommon and osseous disease generally occurs in the context of non-osseous or disseminated Kaposi sarcoma [83]. Its pathogenesis is virus-driven rather than defined by the recurrent somatic mutations or gene fusions characteristic of several other vascular neoplasms discussed above, and nuclear HHV-8 latent nuclear antigen (LANA) expression provides an important diagnostic marker [84]. Recognition of skeletal Kaposi sarcoma is nevertheless relevant to the differential diagnosis of vascular lesions involving bone and further illustrates the biological diversity of lesions capable of producing an osseous vascular phenotype.

Collectively, these findings expand the molecular landscape of vascular lesions involving bone beyond the principal WHO-defined primary bone vascular neoplasms. They demonstrate that superficially related osseous vascular phenotypes may arise through fundamentally different mechanisms, including recurrent gene fusions, PI3K–AKT–mTOR and RAS–MAPK pathway activation, Gαq signaling abnormalities, and virus-associated oncogenesis. Molecular findings should therefore be integrated with morphology, immunophenotype, anatomical distribution, radiological features, and clinical context, particularly when distinguishing true vascular neoplasms from vascular malformations and other vascular lesions involving bone.

The principal molecular alterations and their diagnostic, biological, and potential translational significance are summarized in Table 4.

### 5.2. Fusion-Driven Endothelial Tumor Biology

The discovery of recurrent gene fusions has fundamentally transformed the understanding of endothelial neoplasia, demonstrating that morphologically related vascular tumors may arise through distinct molecular mechanisms rather than representing different stages of a common biological process. In primary vascular tumors of bone, recurrent fusions are not merely diagnostic biomarkers but represent major oncogenic events defining specific entities. These alterations generate aberrant transcriptional regulators that perturb endothelial transcriptional programs, providing mechanistic insights that extend beyond tumor classification [23,35,55,61]. The following discussion focuses on the canonical fusion-driven mechanisms of epithelioid hemangioma and epithelioid hemangioendothelioma; additional fusion-defined vascular neoplasms involving bone, including NFATC1/NFATC2-rearranged neoplasms and pseudomyogenic hemangioendothelioma, are summarized in Section 5.1 and Table 4.

#### 5.2.1. FOS/FOSB-Driven Endothelial Tumorigenesis

Epithelioid hemangioma (EH) is characterized in molecular subsets by recurrent *FOS* and *FOSB* rearrangements. The identification of these alterations provided molecular evidence supporting the neoplastic nature of EH and established recurrent AP-1 family alterations as an important component of its molecular pathogenesis. Multiple rearrangements involving *FOS* and *FOSB* have subsequently been identified, including *FOS::LMNA*, *FOS::VIM*, *ZFP36::FOSB*, and *WWTR1::FOSB*, thereby expanding the molecular spectrum of EH [35,36,85].

*FOS* and *FOSB* encode components of the activator protein-1 (AP-1) transcription complex, a key regulator of endothelial proliferation, differentiation, and stress-responsive transcription. Available evidence indicates that these rearrangements result in aberrant expression and prolonged transcriptional activity of FOS-family proteins. However, unlike EHE, their downstream functional consequences remain only partially characterized. Current evidence therefore supports *FOS/FOSB* rearrangements as central molecular drivers of EH, whereas the downstream transcriptional programs responsible for its characteristic morphology and biological behavior remain incompletely understood [35,36].

#### 5.2.2. Hippo Pathway Dysregulation in Epithelioid Hemangioendothelioma

Conventional epithelioid hemangioendothelioma (EHE) is characterized in the great majority of cases by the *WWTR1::CAMTA1* fusion, one of the most reproducible disease-defining genomic alterations in vascular pathology [1,55]. Unlike *FOS/FOSB*-driven EH, the biological consequences of this fusion have been investigated in experimental models, providing direct evidence for its role as an oncogenic driver.

*WWTR1* encodes TAZ, a major downstream transcriptional effector of the Hippo signaling pathway. Under physiological conditions, Hippo signaling restricts YAP/TAZ activity through phosphorylation-dependent cytoplasmic sequestration and degradation, thereby contributing to the regulation of tissue growth and cellular homeostasis. TAZ::CAMTA1 escapes normal Hippo-dependent regulation and drives aberrant TEAD-dependent transcription. Functional studies have demonstrated that TAZ::CAMTA1 expression in endothelial cells is sufficient to induce vascular tumors recapitulating key features of EHE and that disruption of the TAZ::CAMTA1–TEAD interaction inhibits transformation, establishing the fusion as a principal driver of EHE tumorigenesis [61].

Further mechanistic studies have demonstrated that TAZ::CAMTA1 interacts with components of the Ada2a-containing histone acetyltransferase (ATAC) complex, linking fusion-driven transcriptional activity to chromatin regulation [60]. These findings indicate that EHE pathogenesis involves coordinated transcriptional and chromatin-regulatory dysregulation rather than perturbation of a single downstream signaling output. Nevertheless, the molecular determinants underlying the marked clinical heterogeneity observed among patients with genetically similar tumors remain incompletely understood.

#### 5.2.3. YAP1::TFE3 Defines a Distinct Biological Subtype

A minority of EHEs harbor the *YAP1::TFE3* fusion, defining a genetically and morphologically distinct molecular subtype. Compared with conventional EHE, these tumors typically exhibit more conspicuous vasoformation, abundant eosinophilic cytoplasm, and strong nuclear TFE3 expression, supporting their recognition as a distinct fusion-defined subgroup [54,59].

Although *YAP1::TFE3* and *WWTR1::CAMTA1* involve the paralogous Hippo pathway effectors YAP and TAZ, respectively, the resulting fusion proteins are not biologically equivalent. Functional studies indicate partial convergence of TAZ::CAMTA1- and YAP::TFE3-driven transcriptional programs, including interaction with the ATAC chromatin-regulatory complex, while also demonstrating distinct transcriptional outputs associated with each fusion [60].

Collectively, these fusion-driven mechanisms establish distinct transcriptional programs underlying the biological diversity of EH and EHE. Their characterization provides a molecular framework in which histologically related vascular neoplasms can arise through different oncogenic mechanisms. Important unresolved questions include the determinants of tissue specificity and clinical heterogeneity, as well as the mechanisms through which fusion-driven transcriptional programs interact with the local tissue microenvironment.

### 5.3. Genomic Complexity of Primary Bone Angiosarcoma

Unlike epithelioid hemangioma and epithelioid hemangioendothelioma, primary bone angiosarcoma (B-AS) is not characterized by a recurrent disease-defining fusion but instead exhibits a genetically heterogeneous landscape involving multiple genomic alterations. This distinction supports a separate model of endothelial tumorigenesis characterized by marked intertumoral heterogeneity and genomic complexity [47,80,81,82].

Molecular data specifically addressing primary B-AS remain limited because of its exceptional rarity. A recent bone-specific genomic series identified *KDR* mutations and *MYC* amplification among the recurrent molecular alterations, together with an overall low tumor mutational burden, while no single molecular event emerged as disease-defining [47]. These recent findings extend earlier bone-specific genomic observations demonstrating substantial intertumoral heterogeneity and distinct genomic profiles in primary angiosarcoma of bone, including MYC amplification in a subset of cases [57]. Importantly, MYC amplification is substantially enriched in secondary angiosarcomas, particularly those associated with prior radiation exposure or chronic lymphedema, whereas FLT4 amplification has been reported predominantly in secondary angiosarcoma and may occur in association with MYC amplification [56,58]. Thus, although MYC amplification can occur in primary B-AS, MYC and FLT4 alterations should not be interpreted as universal or disease-defining molecular features of primary osseous angiosarcoma.

Broader genomic analyses of angiosarcoma have identified recurrent alterations affecting angiogenic and intracellular signaling pathways, including *PTPRB* and *PLCG1* mutations and alterations involving components of the RAS/MAPK pathway, supporting roles for dysregulated angiogenic and intracellular signaling in angiosarcoma pathogenesis [80,81,82]. More recent large-scale registry-based genomic analysis has additionally identified recurrent alterations involving TP53, KDR, PIK3CA, MYC, and FLT4, further illustrating the genomic heterogeneity of angiosarcoma [86].

Importantly, these findings derive predominantly from non-osseous angiosarcomas and should therefore be interpreted as broader mechanistic context rather than as established molecular features of primary bone disease.

**Figure 2:**
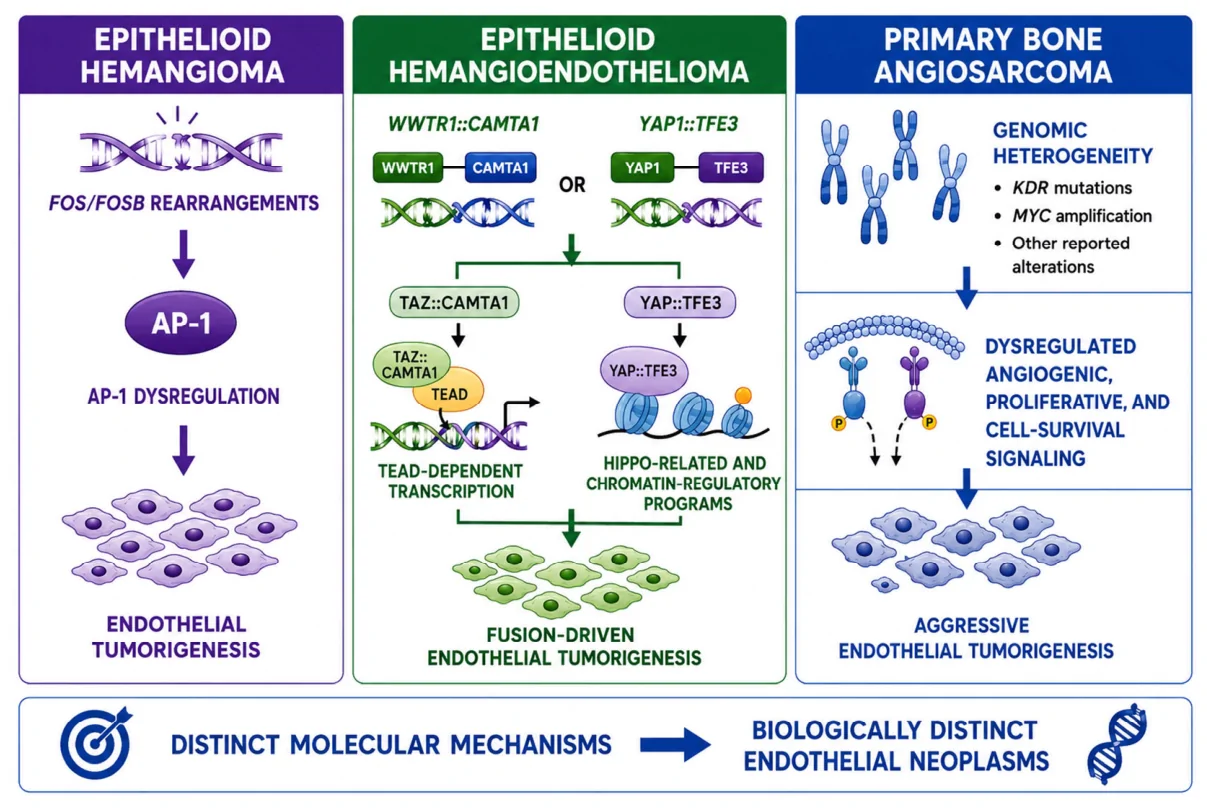
Molecular pathogenesis of primary bone vascular neoplasms. The schematic summarizes the principal molecular mechanisms associated with selected primary bone vascular neoplasms. Recurrent *FOS/FOSB* rearrangements characterize molecular subsets of epithelioid hemangioma, whereas the *WWTR1::CAMTA1* and *YAP1::TFE3* fusions define conventional and distinct fusion-associated subsets of epithelioid hemangioendothelioma, respectively. In contrast, primary bone angiosarcoma exhibits a heterogeneous genomic landscape without a single disease-defining alteration. These molecular differences illustrate biologically distinct mechanisms of endothelial tumorigenesis and provide a basis for investigating tumor–microenvironment interactions. Solid arrows indicate the direction of the depicted molecular or biological processes, whereas dashed arrows indicate proposed or non-linear associations between heterogeneous molecular alterations and downstream signaling effects [35,36,47,54,55,85].

Recent large-scale multi-omic analyses further demonstrate that angiosarcoma comprises biologically distinct molecular subgroups shaped, at least in part, by anatomical site and etiological context. Integrated transcriptomic, somatic, germline, and immunoprofiling approaches have revealed subgroup-specific molecular programs and driver alterations, further emphasizing the biological heterogeneity of angiosarcoma [21,87]. More recently, a patient-partnered multi-omic analysis of 254 patients further demonstrated marked site-associated molecular heterogeneity, identifying distinct transcriptional and genomic programs across angiosarcoma subtypes, with differential involvement of KDR, PLCG1, POT1, MYC, and FLT4 [21]. These findings extend earlier multiomic studies demonstrating molecularly and immunologically distinct angiosarcoma subtypes [87]. Although these observations should not be extrapolated directly to primary bone angiosarcoma, they provide a broader mechanistic framework and reinforce the need to investigate B-AS independently in adequately annotated bone-specific cohorts. Future collaborative studies integrating genomic, transcriptomic, and other complementary molecular approaches are needed to define bone-specific molecular subtypes and clarify the biological basis of tumor heterogeneity [1,82,87]. The distinct molecular mechanisms underlying epithelioid hemangioma, epithelioid hemangioendothelioma, and primary bone angiosarcoma are summarized in Figure 2.

## 6. The Bone Microenvironment: Angiogenesis and Vascular–Bone Crosstalk

Primary vascular neoplasms arising within bone develop within a highly specialized tissue environment shaped by continuous and tightly coordinated skeletal remodeling. Endothelial cells, osteoblasts, osteoclasts, mesenchymal stromal cells, and osteocytes form an integrated multicellular network that contributes to skeletal homeostasis [11,12,88,89].

Primary bone vascular neoplasms should therefore be considered not only in relation to their intrinsic molecular alterations but also within the specialized skeletal niche in which neoplastic endothelial cells coexist with vascular, stromal, and bone-resident components. Although direct mechanistic evidence in primary bone vascular neoplasms remains limited, established principles from bone and vascular biology provide a conceptual framework for investigating how the skeletal microenvironment may influence tumor behavior [11,12,88,89,90].

### 6.1. Vascular–Bone and Stromal Crosstalk

A defining feature of the skeletal microenvironment is the intimate coupling between angiogenesis and osteogenesis, two biologically coordinated processes that support bone growth, remodeling, and repair. This concept has reshaped the understanding of bone biology by demonstrating that blood vessels actively contribute to skeletal homeostasis rather than merely supplying oxygen and nutrients [11,88,89]. The discovery of specialized type H endothelial vessels (CD31^hi^EMCN^hi^), closely associated with osteoprogenitor cells, established that distinct endothelial subpopulations contribute to bone formation through functional interactions with osteogenic cells [11,88,89]. Experimental studies have implicated interconnected signaling pathways, including VEGF, Notch, and HIF-related signaling, in the coordination of endothelial and osteogenic responses within the physiological skeletal niche [11,88,89].

Although these physiological mechanisms have not been directly demonstrated in primary bone vascular neoplasms, they provide an important conceptual framework for interpreting the biological context of endothelial tumors arising within the skeleton. Primary bone vascular neoplasms develop within a specialized skeletal vascular niche in which neoplastic endothelial cells coexist and may interact with osteogenic and stromal compartments. Whether neoplastic endothelial cells retain, disrupt, or exploit physiological angiogenic–osteogenic programs during tumor progression remains unknown and represents an important area for future investigation.

Beyond vascular specialization, the skeletal microenvironment is characterized by continuous bidirectional communication among endothelial, stromal, and skeletal cells, creating a dynamic regulatory network [12]. Endothelial cells themselves exhibit substantial tissue-specific heterogeneity and functional plasticity, with their phenotypic and transcriptional states shaped by local cellular and microenvironmental cues [90]. Osteoblasts, osteoclasts, mesenchymal stromal cells, and osteocytes communicate through growth factors, cytokines, extracellular matrix components, and direct cell–cell interactions to coordinate bone remodeling [9,12,91]. Endothelial cells reciprocally participate in the regulation of osteogenic differentiation and skeletal homeostasis [11,88,89].

An instructive paradigm is provided by giant cell tumor of bone (GCTB), in which *H3F3A*-mutant neoplastic stromal cells coexist with an osteoclast-rich microenvironment involving RANK–RANKL signaling [92,93]. Although biologically distinct from vascular bone neoplasms, GCTB illustrates how tumor-intrinsic molecular alterations can coexist with profound remodeling of the skeletal cellular microenvironment [92,93].

The extracellular matrix further contributes to this network by providing structural and biomechanical context while serving as a reservoir of bioactive molecules. In particular, TGF-β1 and IGF-1 stored within the bone matrix can be released during osteoclastic bone resorption and participate in the coupling of bone resorption and formation [91,94,95]. Such matrix-derived signals contribute to the local regulatory environment encountered by endothelial, stromal, and skeletal cells. Although the specific cellular interactions underlying primary bone vascular neoplasms remain largely unexplored, these principles support the hypothesis that neoplastic endothelial cells may be exposed to stromal and extracellular matrix-derived signals capable of influencing tumor adaptation and progression [12,96].

### 6.2. Mechanobiology of the Bone Microenvironment

An additional regulatory dimension of the skeletal microenvironment is provided by mechanobiology. Bone is continuously exposed to mechanical forces and changes in extracellular matrix properties, while endothelial cells respond to biomechanical cues through mechanosensitive signaling pathways [97]. Consistent with this mechanosensitive behavior, experimental studies in human bone-regenerative settings have shown that matrix stiffness and mechanical strain can modulate local growth-factor signaling involving TGF-β1 and IGF-1, thereby influencing osteogenic differentiation [98].

Among the molecular mediators of vascular mechanotransduction, the Hippo pathway effectors YAP and TAZ have emerged as important regulators linking mechanical cues to endothelial transcriptional responses and vascular homeostasis [97]. This is particularly relevant to epithelioid hemangioendothelioma, in which the disease-defining *WWTR1::CAMTA1* and *YAP1::TFE3* fusions involve *WWTR1* and *YAP1*, which encode the Hippo pathway effectors TAZ and YAP, respectively [54,60,61]. The convergence of fusion-driven YAP/TAZ dysregulation in EHE with the mechanosensitive functions of YAP/TAZ in vascular biology raises the possibility that the biomechanical properties of the skeletal niche may modulate the behavior of fusion-driven endothelial tumor cells [54,60,61,97].

However, current evidence remains indirect, and it is unknown whether bone stiffness, matrix architecture, mechanical loading, or other biomechanical features directly modulate fusion-driven transcriptional programs or tumor behavior in primary bone vascular neoplasms. This potential relationship should therefore be regarded as a biologically plausible hypothesis requiring experimental validation rather than an established pathogenic mechanism.

### 6.3. Biological Implications and Knowledge Gaps

Collectively, these observations support a conceptual shift from viewing bone as a passive anatomical site to considering the skeletal microenvironment as a potentially active modulator of endothelial tumor biology. However, direct disease-specific evidence remains limited, and many of these concepts require validation in experimental models specifically addressing primary bone vascular neoplasms.

Future studies integrating molecular pathology with functional approaches, single-cell transcriptomics, and spatial profiling of the skeletal niche may help determine how vascular–bone interactions contribute to tumor heterogeneity, progression, and therapeutic response [17,19,20]. These considerations also provide a biological rationale for examining the immune component of the skeletal tumor microenvironment [9,14,15,16].

## 7. Osteoimmunology of Primary Bone Vascular Neoplasms

### 7.1. Osteoimmune and Immune–Endothelial Crosstalk

The specialized bone microenvironment described in the previous section is regulated not only by vascular and skeletal cells but also by an integrated immune network that contributes to bone remodeling and is closely interconnected with vascular and stromal signaling [9,12,14,15]. This dynamic interaction between the skeletal and immune systems, broadly encompassed by the concept of osteoimmunology, involves reciprocal communication among immune and bone-resident cells through cytokines, chemokines, growth factors, and direct cellular interactions [9,12,14].

Immune and endothelial cells engage in bidirectional signaling within the tumor microenvironment, linking vascular regulation with local immune responses [12,13,16]. Resident and infiltrating immune populations, including macrophages, other myeloid populations, and T lymphocytes, can modulate angiogenesis and endothelial function through mediators including VEGF, TGF-β, TNF-α, and multiple interleukins [12,13,16]. Conversely, endothelial cells can influence immune-cell recruitment, vascular permeability, and local cytokine and chemokine gradients, thereby contributing to the spatial organization and functional state of the immune microenvironment [12,13,16].

Osteoimmune signaling also directly intersects with skeletal remodeling. The RANK/RANKL/OPG axis provides a central molecular link between immune regulation and osteoclast differentiation, while inflammatory cytokines and other immune-derived mediators can modulate both osteoclast and osteoblast activity [9,12,14,15,16]. Together, these mechanisms provide a biological interface through which immune activation, vascular remodeling, and bone turnover may become functionally interconnected within the skeletal niche [9,12,14,15,16].

Although these mechanisms are well established in physiological bone remodeling, inflammatory skeletal disease, and broader tumor biology, their specific contribution to primary bone vascular neoplasms remains poorly defined [9,12,14,15,16]. The coexistence of neoplastic endothelial cells, specialized skeletal vasculature, bone-resident cells, and immune populations nevertheless provides a biological rationale for investigating whether neoplastic endothelial cells exploit or reshape osteoimmune networks during tumor development and progression. At present, however, immune–endothelial and immune–bone interactions in these neoplasms should be regarded as biologically plausible frameworks rather than confirmed disease-specific pathogenic mechanisms.

### 7.2. Translational Implications and Knowledge Gaps

Characterization of the immune microenvironment may provide new insights into the biological heterogeneity, progression, and therapeutic response of primary bone vascular neoplasms. In particular, defining macrophage states, lymphocyte infiltration, immune–endothelial signaling, and their spatial relationships with tumor and bone-resident cells could help identify distinct immune–vascular ecosystems with potential biological or clinical relevance [13,16,17].

**Figure 3:**
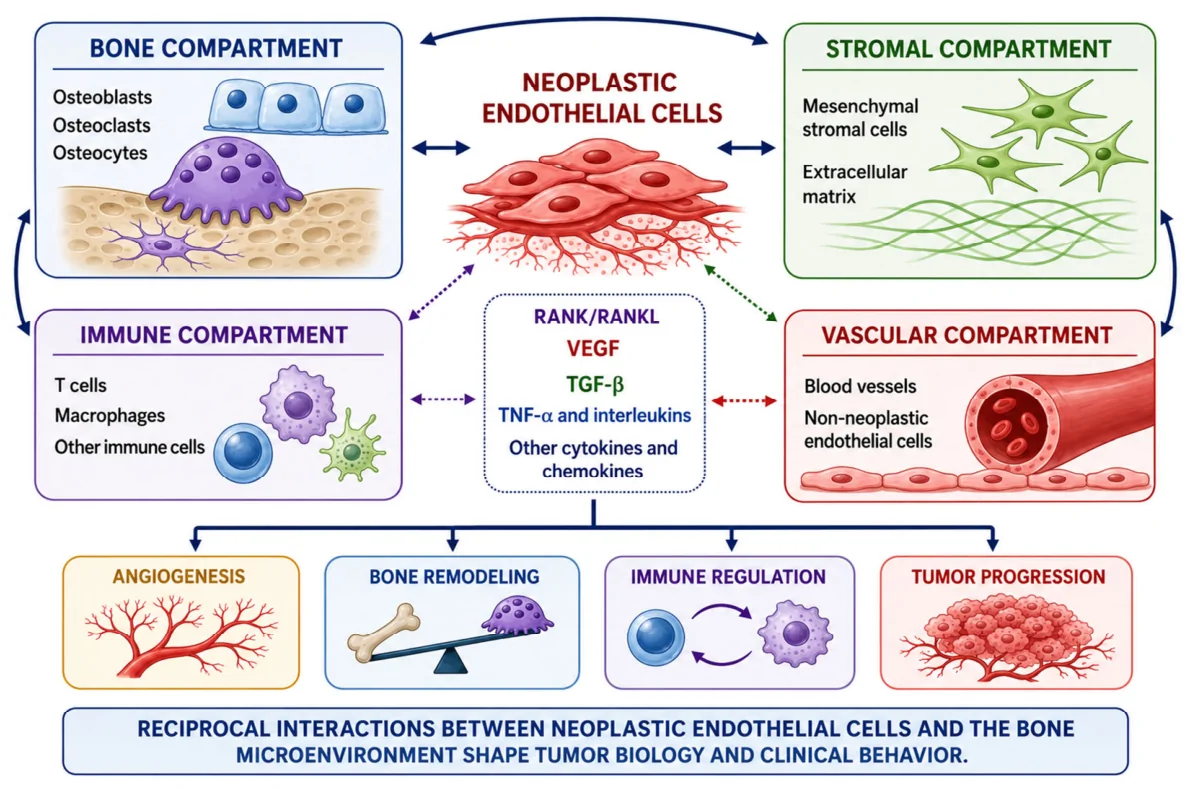
The primary bone vascular tumor ecosystem: integrated vascular–bone–immune crosstalk. The conceptual model illustrates potential reciprocal interactions among neoplastic endothelial cells, bone-resident cells, mesenchymal stromal cells, immune populations, non-neoplastic vascular endothelial cells, and the extracellular matrix within the skeletal tumor microenvironment. Established principles of bone biology, vascular biology, and osteoimmunology support interconnected signaling through RANK/RANKL, VEGF, TGF-β, TNF-α, interleukins, and other cytokines and chemokines, with potential effects on angiogenesis, bone remodeling, immune regulation, and tumor progression. However, the precise organization and functional contribution of these interactions in primary bone vascular neoplasms remain incompletely defined and require disease-specific experimental validation. Solid bidirectional arrows indicate reciprocal intercompartmental interactions, dotted arrows denote mediator-associated or paracrine signaling, and branching arrows connect the integrated network with its proposed biological outcomes [9,12,15,16].

Future studies integrating histopathology and molecular pathology with single-cell transcriptomic and spatial profiling approaches will be required to determine whether such ecosystems characterize specific tumor entities or biological states and whether they have prognostic or therapeutic significance [12,13,16,17,19,20]. The proposed vascular–bone–immune interactions and their potential biological consequences are summarized conceptually in Figure 3.

## 8. Biomarkers, Molecular Diagnostics, and Precision Oncology

### 8.1. Integrated Diagnostic Biomarkers

Precision oncology remains at an early stage in primary bone vascular neoplasms because of their extreme rarity, molecular heterogeneity, and the scarcity of prospective disease-specific studies. Although molecular biomarkers are increasingly incorporated into their biological and clinical characterization, their prognostic and predictive utility remains considerably less well validated [64,99,100].

Molecular biomarkers have their most established clinical role in the diagnostic classification of selected primary bone vascular neoplasms, with molecular techniques increasingly complementing morphology and immunohistochemistry in diagnostically challenging bone and soft-tissue tumors [99,100]. In epithelioid hemangioendothelioma, detection of the canonical *WWTR1::CAMTA1* fusion or, less frequently, the *YAP1::TFE3* fusion provides highly specific molecular support for diagnosis and identifies genetically defined molecular subsets [54,55,64,101]. Nuclear CAMTA1 expression provides a useful immunohistochemical surrogate for conventional EHE, whereas TFE3 immunoreactivity may support recognition of the *YAP1::TFE3*-associated subset but requires cautious interpretation because it is not entirely specific [18,63,101,102]. FISH and RNA-based molecular assays can directly demonstrate the corresponding rearrangements or fusion transcripts and may be particularly valuable in morphologically or immunophenotypically challenging cases [18,99,101,102].

In epithelioid hemangioma, recurrent *FOS* and *FOSB* rearrangements provide molecular support for diagnosis in selected morphologically challenging cases, although these alterations should always be interpreted within the appropriate morphological and clinicopathological context [35,36,85]. Additional molecular alterations may provide diagnostically informative support in selected vascular neoplasms involving bone. These include NFATC1/NFATC2 rearrangements in an emerging group of predominantly intraosseous vascular neoplasms, recurrent IDH1/IDH2 mutations in spindle cell hemangioma, and FOSB-associated rearrangements in pseudomyogenic hemangioendothelioma. As with other molecular findings in this spectrum, these alterations should be interpreted in conjunction with morphology, anatomical distribution, and clinicoradiological context rather than as isolated diagnostic markers.

In contrast, primary bone angiosarcoma lacks a single entity-defining molecular alteration. Its diagnosis therefore continues to depend on the integration of morphology, immunophenotypic findings, and clinicoradiological features, with molecular profiling reserved for selected cases in which additional genomic characterization or exclusion of relevant molecular mimics is diagnostically informative [3,5,47].

### 8.2. Emerging Molecular Biomarkers and Biological Stratification

Beyond entity-defining gene fusions, next-generation sequencing (NGS), RNA sequencing, and other high-throughput molecular approaches are expanding the molecular characterization of vascular neoplasms by enabling broader assessment of genomic alterations and transcriptional programs [64,100,103,104].

Broader molecular profiling may also provide valuable biological information in genetically heterogeneous malignant vascular tumors, including angiosarcoma [103,104,105]. Transcriptomic, single-cell, and spatial molecular approaches offer additional opportunities for identifying biologically distinct tumor states and microenvironmental features that may not be fully captured by morphology or individual genomic alterations [17,19,20]. However, their application specifically to primary bone vascular neoplasms remains limited by small cohorts, restricted tissue availability, and the absence of disease-specific validation.

Future integration of histopathological, genomic, transcriptomic, spatial, and microenvironmental information may enable multidimensional biological stratification. At present, however, such approaches should be regarded primarily as research and biomarker-discovery strategies rather than established components of routine clinical management [19,20,103,104,105].

### 8.3. From Molecular Profiling to Precision Oncology

The transition from molecular diagnosis to precision oncology remains one of the major challenges in primary bone vascular neoplasms. Although molecular profiling can refine diagnostic classification and reveal potentially relevant biological pathways, relatively few molecular alterations currently have validated predictive significance or directly determine systemic treatment.

This distinction is particularly important for tumors characterized by recurrent gene fusions or rearrangements. Alterations involving *FOS/FOSB* in EH and *WWTR1::CAMTA1* or *YAP1::TFE3* in EHE have substantial diagnostic and biological significance, but no validated therapies directly targeting these fusion-associated alterations are currently established [35,54,55,61,85]. Recent experimental evidence further suggests that AMPK signaling may represent a potential therapeutic vulnerability in EHE, with AMPK activation suppressing EHE cell growth through modulation of TAZ–CAMTA1 activity and inhibition of mTOR signaling [106].

In EHE, systemic treatment remains individualized, with anti-angiogenic and mTOR-directed approaches among the strategies used in selected patients with progressive disease [107,108,109]. Importantly, these therapeutic data derive predominantly from EHE cohorts that are not restricted to primary bone disease and should therefore not be interpreted as bone-specific evidence.

In angiosarcoma, broader genomic profiling may identify potentially actionable alterations in selected cases; however, molecular heterogeneity and the predominance of evidence from non-osseous disease currently limit the establishment of bone-specific predictive biomarkers [21,82,87]. Similarly, immune checkpoint inhibition has demonstrated clinical activity in subsets of angiosarcoma, but its precise role in primary bone angiosarcoma remains undefined [110,111].

Molecular findings should currently complement, rather than replace, integrated clinicopathological assessment and multidisciplinary sarcoma management. Current and emerging therapeutic approaches, together with their principal clinical contexts, evidence base, and major limitations, are summarized in Table 5.

**Figure 4:**
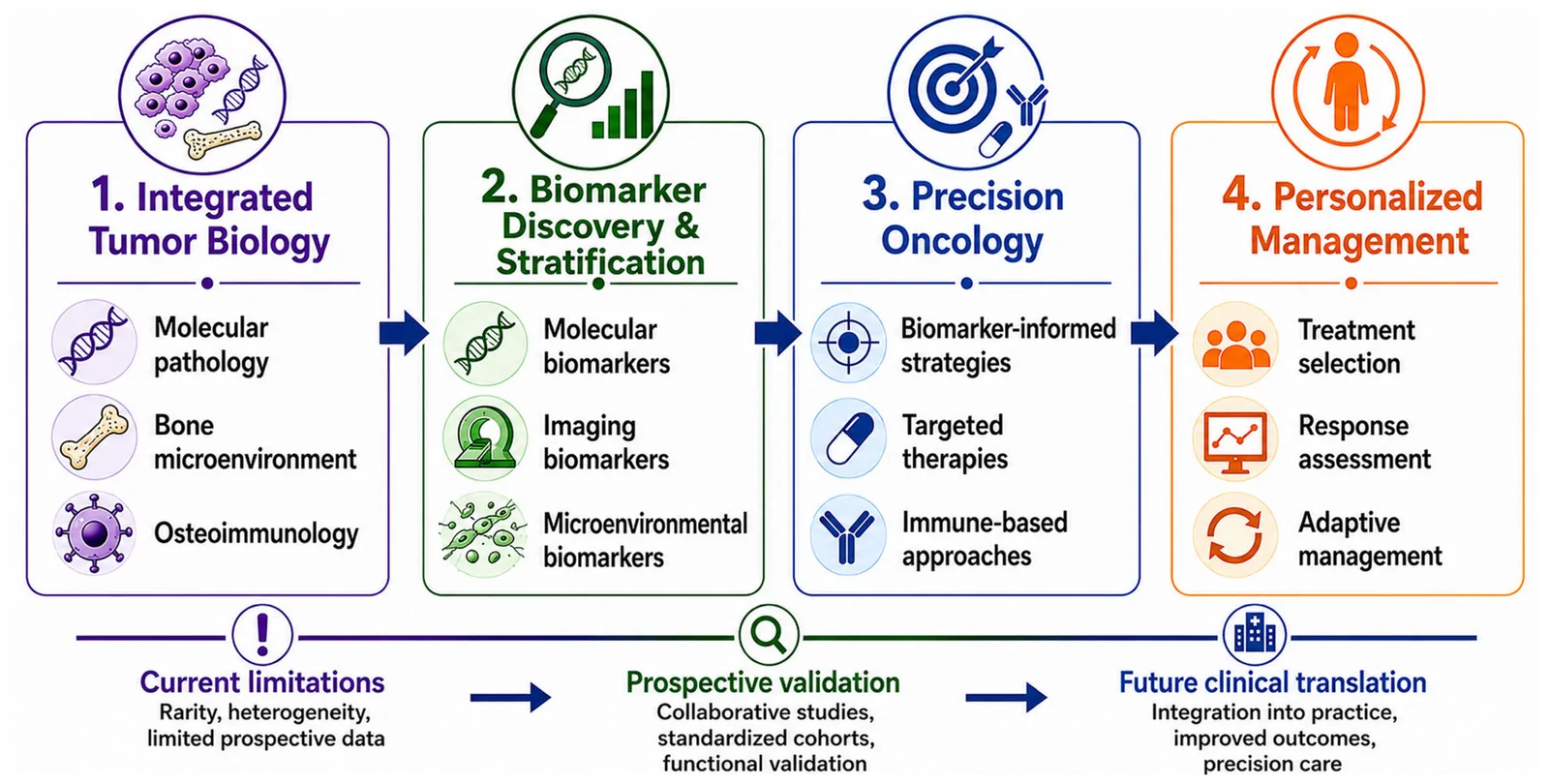
From Tumor Biology to Precision Oncology in Primary Bone Vascular Neoplasms. Integrative framework linking tumor-intrinsic molecular alterations with the bone microenvironment and osteoimmune interactions, and illustrating their potential integration into biomarker discovery, biological stratification, and emerging precision-oncology strategies. Disease-specific validation and prospective clinical investigation remain necessary before these approaches can support individualized patient management [12,14,64,100,103,105].

Collectively, these advances define a translational continuum from molecular classification to biomarker-informed patient stratification. However, the transition from biological insight to clinically validated precision oncology remains incomplete and represents a major priority for future research (Figure 4).

## 9. Future Perspectives

Despite substantial advances in the histopathological and molecular characterization of primary bone vascular neoplasms, their extreme rarity continues to limit biological understanding, experimental modeling, biomarker validation, and prospective clinical investigation. Future research should move beyond molecular characterization alone to determine how tumor-intrinsic alterations interact with the specialized skeletal microenvironment to influence disease initiation, progression, and therapeutic response.

Although tumor-intrinsic molecular mechanisms are increasingly well defined in selected entities, particularly fusion-driven transcriptional dysregulation in EHE [60,61], their interaction with the skeletal microenvironment remains largely unresolved. Key disease-specific questions include whether the mechanical and extracellular matrix properties of bone modulate fusion-driven oncogenic transcriptional programs; whether distinct stromal, osteoclast, osteoblast, or immune-cell states characterize individual vascular tumor entities; and whether the skeletal niche contributes to biological differences between osseous and non-osseous vascular neoplasms sharing related molecular alterations. Addressing these questions will be important for distinguishing established tumor-intrinsic mechanisms from microenvironmental interactions that remain hypothesis-generating.

Advanced experimental systems represent an important priority. Patient-derived organoids, three-dimensional co-culture systems, and other physiologically relevant models could provide platforms for investigating endothelial–stromal–bone interactions and evaluating candidate therapeutic vulnerabilities. Although disease-specific organoid models for primary bone vascular neoplasms remain to be established, incorporation of endothelial, stromal, immune, and bone-resident components may ultimately enable more faithful reconstruction of the skeletal tumor ecosystem and facilitate functional validation of molecular findings [112].

A complementary priority is the development of multidimensional molecular maps through integration of genomic, transcriptomic, epigenomic, proteomic, single-cell, and spatial approaches. Emerging regulatory layers, including non-coding RNAs such as circular RNAs, may further expand this molecular framework by providing candidate biomarkers and potential modulators of tumor–immune interactions, although their relevance to primary bone vascular neoplasms remains unexplored [113]. Rather than generating descriptive molecular datasets alone, future studies should link molecular and cellular states to histopathological features, anatomical context, clinical behavior, and therapeutic response.

Such integrated analyses may help define biologically meaningful disease subsets, identify candidate biomarkers, and distinguish disease-specific mechanisms from features shared more broadly across vascular neoplasms. Computational pathology and artificial intelligence may further facilitate integration of histological, radiological, molecular, and clinical information. However, development of robust disease-specific algorithms will require multicenter datasets, standardized annotation, external validation, and international data-sharing frameworks rather than isolated single-institution models [114,115].

Liquid biopsy approaches, including circulating tumor DNA and other blood-based molecular analytes, may provide opportunities for minimally invasive molecular profiling and longitudinal disease monitoring. However, evidence specific to primary bone vascular neoplasms is currently lacking, and their feasibility, analytical sensitivity, and clinical utility in these diseases remain to be established. Such approaches should therefore be evaluated prospectively alongside tissue-based molecular profiling rather than extrapolated directly from experience in more common malignancies [116,117,118,119,120].

Improved characterization of the immune microenvironment may also expand future therapeutic opportunities. Identification of tumor-associated antigens and functionally relevant immune–endothelial interactions could provide a biological rationale for exploring antigen-directed or adoptive cellular immunotherapies. Although engineered T-cell approaches are advancing in selected sarcoma subtypes, their relevance to primary bone vascular neoplasms remains entirely investigational. Future studies will therefore need to define suitable target antigens, determine how the skeletal tumor microenvironment influences immune-cell trafficking, persistence, and therapeutic activity, and establish disease-specific preclinical models before such strategies can be meaningfully translated into clinical investigation [121,122].

Ultimately, progress will depend on coordinated international collaboration. Harmonized clinicopathological registries, high-quality biobanks, standardized molecular methodologies, and multicenter translational studies will be essential for generating sufficiently large and well-annotated cohorts to validate biomarkers, develop representative experimental models, and evaluate emerging therapeutic strategies. For tumors encountered only rarely at individual institutions, international rare-tumor networks are not merely advantageous but fundamental to meaningful biological and clinical investigation.

## 10. Conclusions

Primary bone vascular neoplasms represent a biologically heterogeneous and diagnostically challenging group of skeletal tumors. Advances in molecular pathology have substantially refined their classification by demonstrating that morphologically related lesions may represent distinct biological entities characterized by specific molecular alterations, divergent pathogenetic mechanisms, and variable clinical behavior. Integration of molecular findings with conventional histopathology has therefore improved diagnostic precision while providing deeper insight into endothelial tumorigenesis.

Beyond tumor-intrinsic molecular alterations, these neoplasms should also be considered within the specialized skeletal microenvironment in which they arise. Interactions among neoplastic endothelial cells, bone-resident and stromal populations, extracellular matrix components, and immune cells provide an additional layer of biological complexity that cannot be fully captured by genomic alterations alone. Although direct disease-specific evidence remains limited, integrating molecular pathology with bone biology, angiogenesis, and osteoimmunology provides a broader framework for investigating tumor heterogeneity, progression, and therapeutic response.

Together, these perspectives support an integrated biological framework that connects histopathology and molecular classification with the vascular–bone–immune microenvironment. Further disease-specific investigation will be essential to determine its biological and clinical relevance in primary bone vascular neoplasms.

## Figures and Tables

**Table 1:** Comparison of the WHO 2020 and ISSVA 2025 Classification Frameworks in the Context of Primary Bone Vascular Neoplasms.

Feature	WHO 2020 Classification [1,24]	ISSVA 2025 Classification [25,26,27]
**Primary purpose and scope**	Classification of soft tissue and bone tumors, including primary vascular neoplasms of bone	Classification of vascular anomalies, encompassing vascular tumors, vascular malformations, and potentially unique vascular anomalies
**Organizing principles**	Integrated histopathological, immunophenotypic, molecular, and biological criteria	Clinical, anatomical, developmental, hemodynamic, histopathological, and genetic criteria
**Role of pathology**	Central to tumor diagnosis, classification, and differential diagnosis	Essential for characterizing vascular tumors, but not the sole basis of the broader anomaly framework
**Molecular integration**	Defines or supports selected molecularly characterized tumor entities	Increasingly informs the classification and nomenclature of genetically defined vascular anomalies
**Biological behavior**	Distinguishes benign, intermediate/locally aggressive, and malignant vascular neoplasms	Categorizes vascular tumors according to their biological behavior
**Relevance to primary bone vascular neoplasms**	Principal pathological classification framework	Complementary framework that helps distinguish vascular neoplasms from malformations and other vascular anomalies
**Clinical utility**	Supports integrated diagnosis, prognostic assessment, and therapeutic planning	Supports standardized nomenclature and multidisciplinary evaluation

**Table 2:** Comparative clinicopathological, immunophenotypic, and molecular features of conventional hemangioma of bone and intraosseous venous malformation.

Feature	Conventional Hemangioma of Bone	Intraosseous Venous Malformation (VM)
Contemporary classification	Retained as a benign vascular-tumor/pathology-based designation in the bone-tumor literature, although its relationship to lesions classified as vascular malformations under the ISSVA framework remains incompletely resolved [1,3,44].	Vascular malformation rather than vascular tumor according to the ISSVA framework [25,26]. The 2025 ISSVA glossary states that lesions labelled “intraosseous hemangioma” are most often VMs and are more appropriately designated intraosseous venous malformations.
Biological interpretation	Traditionally interpreted as a benign vascular proliferation; no recurrent disease-defining molecular alteration has established conventional hemangioma of bone as a single molecularly uniform neoplastic entity.	Developmental/malformative abnormality of venous vasculature; molecular subsets are increasingly defined by pathogenic variants affecting venous-development signaling pathways.
Histopathology	Well-formed vascular channels lined by cytologically bland endothelial cells without significant atypia or destructive malignant growth [1,3,44].	Dilated, irregular venous-type vascular spaces lined by bland endothelium; variable smooth-muscle investment and thrombosis may occur. Histological overlap with lesions historically diagnosed as intraosseous hemangioma can be substantial [28,29,30,45].
Endothelial markers	CD31+, ERG+, FLI1+; CD34 commonly positive.	CD31/ERG/CD34 may also be expressed and therefore do not, by themselves, distinguish VM from a benign vascular tumor.
GLUT1	Not established as a defining marker of conventional hemangioma of bone. GLUT1 positivity in an appropriate clinicopathological setting supports an infantile-hemangioma phenotype, but available bone-specific evidence is limited.	Generally GLUT1-negative. However, GLUT1 negativity alone does not establish a diagnosis of VM and must be interpreted with clinical, imaging, and pathological findings [30,31].
D2-40/podoplanin	Not a defining diagnostic marker of conventional hemangioma of bone.	Ordinary venous malformations are not defined by D2-40 positivity. D2-40 is principally informative when lymphatic differentiation is being considered; therefore, negativity does not by itself distinguish conventional hemangioma from VM.
TEK (TIE2)	No established recurrent TEK alteration defining conventional hemangioma of bone.	Somatic activating TEK mutations are established drivers of a major subset of sporadic VMs [38].
PIK3CA	No established recurrent PIK3CA alteration defining conventional hemangioma of bone.	Somatic activating PIK3CA mutations define an additional major molecular subset of VMs, including TEK-negative lesions [39].
Other molecular findings	No recurrent disease-defining molecular alteration currently established. Molecular abnormalities defining other vascular tumors (e.g., FOS/FOSB, IDH1/IDH2, or NFATC-family rearrangements) should not be extrapolated to conventional hemangioma.	Selected genetically distinct intraosseous malformation syndromes also exist, including ELMO2-related primary/familial intraosseous vascular malformation [41,42].
Radiological features	Classical appearances vary according to anatomical site; vertebral lesions may show characteristic trabecular patterns, whereas craniofacial and appendicular lesions may have different appearances. Imaging terminology historically overlaps substantially with “intraosseous hemangioma.”	Typically a slow-flow vascular lesion; imaging assessment should evaluate intraosseous extent, cortical involvement, associated soft-tissue components, vascular characteristics, and, where present, features supporting a venous lesion. Imaging alone may not always resolve historically labelled hemangioma from VM [28,29,30,45].
Anatomical-site considerations	The same historical term has been applied to vertebral, craniofacial/calvarial, jaw, and appendicular lesions, but current evidence does not establish that all such lesions constitute one biologically uniform entity.	Reclassification as VM is particularly well documented for craniofacial and jaw intraosseous lesions. Evidence is less comprehensive across all skeletal sites; therefore, site-specific terminology and clinicopathological correlation remain important.
Diagnostic approach	Diagnosis should integrate morphology, anatomical site, imaging, clinical behavior, and—when the differential diagnosis includes a molecularly defined vascular anomaly—appropriate ancillary studies.	Integrated clinicoradiological, pathological, immunophenotypic, and, when informative, molecular assessment is preferred. Detection of TEK/PIK3CA alterations supports VM biology but must be interpreted in the appropriate lesion context.
Principal limitation	The term remains entrenched in bone pathology and radiology but does not currently correspond to a universally demonstrated molecularly homogeneous entity.	Molecular testing is informative but not universally positive; absence of an identified pathogenic variant does not by itself exclude VM.

**Table 3:** Integrated Clinicopathological, Immunophenotypic, Molecular, and Diagnostic Features of Primary Bone Vascular Neoplasms According to the WHO 2020 Classification.

Entity and WHO Category	Histology	Immunophenotype	Molecular Features	Diagnostic Significance
Hemangioma of bone—benign [1,3,44]	Bland, well-formed vascular channels without destructive growth. Some lesions overlap morphologically with venous malformations.	CD31+, ERG+, FLI1+; CD34 usually positive. GLUT1 is ancillary in selected differential diagnoses and is not a defining marker [30,31,34].	No established defining alteration. TEK and PIK3CA drive subsets of venous malformations [38,39]; these findings cannot be assigned to conventional bone hemangioma without appropriate classification.	Integrate morphology and imaging. “Hemangioma” should not be used as an umbrella term for intraosseous vascular malformations (see the section “Hemangioma of Bone: Terminological and Biological Heterogeneity”).
Epithelioid hemangioma (EH)—intermediate, locally aggressive [3,35]	Lobular or vasoformative architecture with epithelioid endothelial cells and usually mild atypia.	CD31+, ERG+, FLI1+; CD34 variable; keratin may be focally positive.	FOS or FOSB rearrangements in molecular subsets.	Fusion testing may aid distinction from overlapping vascular neoplasms.
Epithelioid hemangioendothelioma (EHE)—malignant [3,46,54,55]	Cords or nests of epithelioid endothelial cells in myxohyaline stroma; intracytoplasmic vacuoles may occur.	CD31+, ERG+, FLI1+; CD34 variable; keratin may be positive.	WWTR1::CAMTA1 in conventional EHE; YAP1::TFE3 in a distinct subset.	CAMTA1 or TFE3 staining and molecular confirmation may assist diagnosis.
Primary bone angiosarcoma—malignant [3,47,50,56,57,58]	Infiltrative channels or solid growth with atypia, endothelial multilayering, mitoses, and variable necrosis.	CD31+, ERG+, FLI1+; CD34 variable; focal keratin possible.	No defining alteration. KDR mutations and MYC amplification occur in selected cases; MYC and FLT4 amplification are more characteristic of secondary angiosarcoma.	Interpret genomic results with anatomical site and etiology; profiling may reveal candidate targets.

**Table 4:** Molecular Landscape of Primary Bone Vascular Neoplasms and Additional Vascular Lesions Involving Bone: Diagnostic and Translational Significance.

Tumor Entity	Principal Molecular Alteration(s)	Core Biological Mechanism	Molecular Surrogate/Diagnostic Utility	Translational or Therapeutic Implications
Hemangioma [1,3] (WHO/pathology-based designation; (see the section “Hemangioma of Bone: Terminological and Biological Heterogeneity”).	No recurrent disease-defining molecular alteration established for conventional hemangioma of bone. The historical diagnostic category may encompass biologically heterogeneous lesions; TEK and PIK3CA alterations are established drivers of venous malformations rather than defining alterations of conventional hemangioma of bone [38,39].	Molecular pathogenesis remains incompletely characterized. No single molecular mechanism has been established, and the designation should not be assumed to represent a molecularly uniform neoplastic entity.	No specific molecular surrogate; diagnosis remains based on morphology and immunophenotypic findings. Molecular findings should be interpreted together with morphology, immunophenotype, imaging, anatomical site, and clinical context.	No established molecularly targeted strategy specific to conventional hemangioma of bone; pathway-directed approaches used for vascular malformations should not be extrapolated without appropriate biological classification.
Epithelioid hemangioma (EH) [28,38,45,49]	Recurrent *FOS* or *FOSB* rearrangements; reported fusions include *FOS::LMNA*, *FOS::VIM*, *ZFP36::FOSB*, and *WWTR1::FOSB*	Dysregulated AP-1 transcriptional activity; *FOS* rearrangements may generate C-terminally truncated proteins with increased stability and prolonged transcriptional activity	Nuclear FOS/FOSB expression may support selected molecular subsets but is not entirely specific; fusion testing is useful in morphologically challenging or discordant cases	Primarily diagnostic at present; AP-1-related therapeutic vulnerabilities remain experimental, and no validated fusion-directed therapy is available
Conventional epithelioid hemangioendothelioma (EHE) [55,60,61]	*WWTR1::CAMTA1* fusion in the great majority of conventional EHEs	TAZ::CAMTA1 escapes normal Hippo pathway regulation and drives aberrant TEAD-dependent transcription and chromatin-regulatory programs	Nuclear CAMTA1 is a useful surrogate marker; detection of *WWTR1::CAMTA1* provides a major disease-defining discriminator from histological mimics	Provides a rationale for investigating Hippo–YAP/TAZ–TEAD and chromatin-regulatory vulnerabilities; no established fusion-directed therapy
*YAP1::TFE3*-associated EHE [54,59,60,63]	*YAP1::TFE3* fusion	Aberrant YAP::TFE3 transcriptional activity involving Hippo-related and chromatin-regulatory programs that partially overlap with, but remain distinct from, TAZ::CAMTA1-driven biology	Nuclear TFE3 expression and loss of YAP1 C-terminal expression are supportive; molecular confirmation is important because TFE3 expression alone is not specific	Potential pathway-directed vulnerabilities remain investigational; no validated fusion-directed therapy
NFATC1/NFATC2-rearranged vascular neoplasms involving bone	*EWSR1::NFATC1*, *EWSR1::NFATC2*, *FUS::NFATC2* and less common fusion partners	Fusion-associated dysregulation of NFATC-family transcriptional programs; biological mechanisms remain incompletely defined	Molecular detection supports diagnosis in the appropriate morphological and radiological context, but NFATC-family rearrangements are not specific for vascular neoplasia	Currently primarily diagnostic; no established fusion-directed therapy [69,70]
Spindle cell hemangioma involving bone	Recurrent *IDH1/IDH2* hotspot mutations, particularly *IDH1* p.R132C	Recurrent IDH1/IDH2-mutant biology; the precise mechanism linking these alterations to vascular tumorigenesis remains incompletely defined	*IDH1/IDH2* mutation testing may provide strong entity-associated molecular support in diagnostically challenging cases	Primarily diagnostic at present; no established molecularly targeted treatment in this setting [37]
Pseudomyogenic hemangioendothelioma (PHE) involving bone	Recurrent *FOSB*-activating fusions, principally *SERPINE1::FOSB* and *ACTB::FOSB*; rarer *FOSB* fusion partners occur	Fusion-driven FOSB/AP-1 transcriptional dysregulation	Nuclear FOSB expression is a useful diagnostic surrogate but is not entirely specific; molecular confirmation may assist difficult cases	Fusion-defined biology provides a framework for pathway-directed investigation; no established fusion-directed therapy [62,76,77,78,79]
Primary bone angiosarcoma (B-AS) [47,80,81,82]	Genomically heterogeneous; *KDR* mutations and *MYC* amplification have been reported in selected primary bone angiosarcomas; *MYC* amplification is substantially enriched in secondary angiosarcoma, whereas *FLT4* amplification is predominantly described in secondary, frequently *MYC*-amplified disease	Dysregulation of angiogenic, proliferative, cell-survival, and genomic-stability programs without a single unifying molecular driver	No disease-defining molecular test or universally specific surrogate; genomic profiling may support characterization and identify potentially actionable alterations	Supports biomarker-driven investigation of anti-angiogenic and pathway-directed treatments; bone-specific predictive biomarkers remain insufficiently validated [56,57,58]
**Entity**	**Principal Molecular Alteration(s)**	**Core Biological Mechanism**	**Molecular Surrogate/Diagnostic Utility**	**Translational or Therapeutic Implications**
GNAQ-altered vascular lesions involving bone	Somatic *GNAQ* alterations have been demonstrated in selected osseous vascular lesions; direct bone-specific evidence for *GNA11* remains limited	Dysregulation of Gαq-mediated signaling	Molecular findings may support biological classification in selected lesions but are not diagnostic of conventional hemangioma of bone	Pathway implications remain investigational in osseous disease [71]
Generalized lymphatic anomaly (GLA) with skeletal involvement	Somatic activating *PIK3CA* mutations	Constitutive PI3K–AKT–mTOR pathway activation	Supports molecular characterization of GLA as a complex lymphatic anomaly rather than a conventional vascular neoplasm	Provides a biological rationale for pathway-directed therapeutic approaches [72]
Gorham–Stout disease (GSD)	Somatic activating *KRAS* variants have been identified in affected tissue	RAS–MAPK activation associated with abnormal lymphatic development and osseous involvement	Molecular findings can support biological characterization but are not required for diagnosis	Provides a mechanistic rationale for MAPK-pathway-directed investigation [73,74]
Venous malformations, including intraosseous venous malformations where appropriately classified	Somatic activating *TEK* mutations are established drivers of sporadic venous malformations [38]. Somatic activating PIK3CA mutations define an additional major molecular subset [39].	Constitutive TIE2 signaling with downstream PI3K–AKT–mTOR activation.	TEK testing supports classification in selected cases; clinical and radiological correlation remains essential.	PI3K–AKT–mTOR pathway inhibition may be considered in selected complex malformations; treatment is individualized.
Kaposi sarcoma involving bone	HHV-8/KSHV-associated oncogenesis	Virus-driven endothelial oncogenesis involving viral and host-cell signaling programs	Nuclear HHV-8 LANA-1 expression provides an important diagnostic marker and assists in distinguishing Kaposi sarcoma from histological vascular mimics; skeletal involvement should not be classified as a conventional primary vascular neoplasm of bone [84]	Management is determined by the clinical form and systemic context of Kaposi sarcoma rather than by a bone-specific molecular target [83]

**Table 5:** Current and Emerging Therapeutic Strategies for Primary Bone Vascular Neoplasms.

Therapeutic Strategy	Main Clinical Context/Relevant Entities	Current Role and Evidence	Key Limitations/Future Priorities
Observation or active surveillance	Asymptomatic lesions appropriately classified as hemangioma of bone; selected clinically stable or indolent EHE	Appropriate for benign lesions without symptoms or structural risk and for carefully selected patients with indolent EHE when immediate intervention is unnecessary [3,46,108]	Requires diagnostic certainty, individualized imaging follow-up, and assessment of symptoms, anatomical site, and skeletal stability
Surgical management	Symptomatic or structurally significant lesions appropriately classified as hemangioma of bone; EH; resectable localized EHE or angiosarcoma	Principal local treatment across the disease spectrum; the procedure is determined by tumor biology, anatomical site, disease extent, and feasibility of adequate local control [3,46,47,49]	Balance required among oncological control, surgical morbidity, skeletal stability, and functional preservation; evidence is predominantly retrospective
Radiotherapy	Selected unresectable or locally advanced disease; individualized postoperative settings; palliation, primarily in EHE or angiosarcoma	Used selectively when surgery is not feasible or when additional local control or symptom relief is required [3,46,47]	Indications are not standardized, and prospective evidence—particularly for primary bone disease—is lacking
Cytotoxic chemotherapy	Advanced, metastatic, or unresectable aggressive disease, primarily angiosarcoma	Systemic treatment option for advanced angiosarcoma, based largely on broader angiosarcoma and sarcoma experience; conventional chemotherapy generally has limited activity in EHE [3,46,47,108]	Heterogeneous responses; no regimen is specifically validated for primary bone angiosarcoma; prospective bone-specific trials are unavailable
Anti-angiogenic or multikinase therapy	Progressive, unresectable, or metastatic EHE; selected advanced angiosarcoma	VEGF/VEGFR-directed and multikinase agents have demonstrated disease control in selected vascular-tumor cohorts; pazopanib may be considered in selected metastatic EHE [3,64,108,109]	Evidence derives from small, retrospective, predominantly non-bone-specific cohorts; predictive biomarkers and optimal patient selection remain undefined
mTOR-pathway inhibition	Progressive EHE requiring systemic treatment	Sirolimus has produced prolonged disease stabilization in selected patients, particularly in the absence of serosal effusions [64,107,108]	No randomized evidence; activity appears limited in patients with serosal effusions; optimal sequencing and bone-specific efficacy remain uncertain
Immune-checkpoint inhibition	Selected advanced or refractory angiosarcoma; investigational in EHE	Clinical activity has been reported in molecularly or clinically selected angiosarcoma subsets, predominantly in non-osseous disease [82,110,111]	No validated predictive biomarker or established indication specifically for primary bone angiosarcoma
Molecularly informed and combination strategies	Molecularly characterized advanced disease; selected angiosarcoma; investigational relevance to EH and EHE	Genomic profiling may identify candidate therapeutic vulnerabilities, while combinations of angiogenic, immune, and other pathway-directed treatments remain investigational [21,82,87,103,105,111]	No validated therapy directly targets *FOS/FOSB*, *WWTR1::CAMTA1*, or *YAP1::TFE3*; optimal combinations, sequencing, toxicity management, and predictive biomarkers remain undefined

Note: Most evidence on systemic therapy for EHE and angiosarcoma derives from mixed-site or predominantly non-osseous cohorts; therefore, these findings should not be considered specifically validated for primary bone disease.

## Data Availability

No new data were created or analyzed in this study. Data sharing is not applicable to this article.

## Abbreviations

The following abbreviations are used in this manuscript:

AP-1	Activator protein 1
ATAC	Ada2a-containing histone acetyltransferase complex
B-AS	Primary bone angiosarcoma
ECM	Extracellular matrix
EH	Epithelioid hemangioma
EHE	Epithelioid hemangioendothelioma
EMCN	Endomucin
FISH	Fluorescence in situ hybridization
GCTB	Giant cell tumor of bone
GLA	Generalized lymphatic anomaly
GSD	Gorham–Stout disease
HIF-1α	Hypoxia-inducible factor 1 alpha
HHV-8	Human herpesvirus 8
IGF-1	Insulin-like growth factor 1
ISSVA	International Society for the Study of Vascular Anomalies
JAK/STAT	Janus kinase/signal transducer and activator of transcription
KLA	Kaposiform lymphangiomatosis
KSHV	Kaposi sarcoma-associated herpesvirus
LANA	Latency-associated nuclear antigen
MAPK	Mitogen-activated protein kinase
mTOR	Mechanistic target of rapamycin
NF-κB	Nuclear factor kappa B
NFATC	Nuclear factor of activated T cells, cytoplasmic
NGS	Next-generation sequencing
OPG	Osteoprotegerin
PBVNs	Primary bone vascular neoplasms
PHE	Pseudomyogenic hemangioendothelioma
PI3K/AKT	Phosphoinositide 3-kinase/protein kinase B
RANK	Receptor activator of nuclear factor κB
RANKL	Receptor activator of nuclear factor κB ligand
TAZ	Transcriptional coactivator with PDZ-binding motif
TEAD	TEA domain transcription factor
TGF-β	Transforming growth factor beta
TNF-α	Tumor necrosis factor alpha
VEGF	Vascular endothelial growth factor
VEGFR2	Vascular endothelial growth factor receptor 2
WHO	World Health Organization
YAP	Yes-associated protein
